# Genetic architecture and cross-platform validation of apolipoprotein A-IV concentrations

**DOI:** 10.1186/s13059-026-04196-7

**Published:** 2026-07-14

**Authors:** Adriana Koller, Florian Schnitzer, Barbara Kollerits, Claudia Lamina, Samuel Moix, Annique Claringbould, Binisha H. Mishra, Haojie Lu, Johanna F. Schachtl-Riess, Lukas Forer, Christian Gieger, Azin Kheirkhah, Terho Lehtimäki, Pedro Marques-Vidal, Sebastian Schönherr, Klaus J. Stark, Reinhard Würzner, Kai-Uwe Eckardt, Iris M. Heid, Maryam Kavousi, Anna Köttgen, Olli Raitakari, Eric J. G. Sijbrands, Annette Peters, Peter Vollenweider, Florian Kronenberg

**Affiliations:** 1 Institute of Genetic Epidemiology, Medical University of Innsbruck, Schöpfstrasse 3, 6020 Innsbruck, Austria; 2 Department of Computational Biology, University of Lausanne, Lausanne, Switzerland; 3 Swiss Institute of Bioinformatics, Lausanne, Switzerland; 4 Department of Internal Medicine, Erasmus University Medical Center, Rotterdam, The Netherlands; 5 Department of Clinical Chemistry, Faculty of Medicine and Health Technology, Fimlab Laboratories and Finnish Cardiovascular Research Center-Tampere, Tampere University, Tampere, Finland; 6 Department of Epidemiology, Erasmus University Medical Center Rotterdam, Rotterdam, The Netherlands; 7 Institute of Epidemiology, Helmholtz Zentrum München, German Research Center for Environmental Health (GmbH), Neuherberg, Germany; 8 Research Unit Molecular Epidemiology, Helmholtz Zentrum München, German Research Center for Environmental Health (GmbH), Neuherberg, Germany; 9 German Center for Diabetes Research (DZD), Neuherberg, Germany; 10 Department of Medicine, Internal Medicine, Lausanne University Hospital (CHUV) and University of Lausanne, Lausanne, Switzerland; 11 Department of Genetic Epidemiology, University of Regensburg, Regensburg, Germany; 12 Institute of Hygiene and Medical Microbiology, Medical University of Innsbruck, Innsbruck, Austria; 13 Department of Nephrology and Hypertension, University Hospital Erlangen, Friedrich-Alexander-Universität Erlangen-Nürnberg, Erlangen, Germany; 14 Department of Nephrology and Medical Intensive Care, Charité – Universitätsmedizin Berlin, Berlin, Germany; 15 Institute of Epidemiology and Prevention, Faculty of Medicine and Medical Center - University of Freiburg, Freiburg, Germany; 16 Centre for Population Health Research, University of Turku and Turku University Hospital, Turku, Finland; 17 Research Centre of Applied and Preventive Cardiovascular Medicine, University of Turku, Turku, Finland; 18 Department of Clinical Physiology and Nuclear Medicine, Turku University Hospital, Turku, Finland; 19 InFLAMES Research Flagship, University of Turku, Turku, Finland

**Keywords:** Apolipoprotein A-IV, GWAS, Lipid traits, Genetic correlation, Kidney function

## Abstract

**Background:**

Apolipoprotein A-IV (apoA-IV) plays key roles in lipid metabolism, reverse cholesterol transport, and kidney function, yet its genetic determinants remain poorly defined. We conduct a genome-wide association study (GWAS) meta-analysis of apoA-IV concentrations measured by ELISA in 25,181 individuals and combine these with proteomic data from 33,995 UK Biobank participants (Olink platform), yielding a total sample of 59,176. We perform genetic correlations and colocalization analyses to explore links with lipid, renal, and other complex traits.

**Results:**

The GWAS identifies several novel loci to be associated with apoA-IV concentrations (*TDRD5*, *DPP4*, *MYL3*, *MORC1*, *MCUB*, *GATA4*, *ZPR1*, *UMOD*, G*LP2R*, *SLC38A10* and *APOE*) besides two previously identified loci (*APOA5-A4-C3-A1* cluster and *KLKB1*). Cross-platform comparison shows strong concordance of effect directions and magnitudes, underscoring the robustness of findings across measurement techniques. Global genetic correlation reveals significant shared genetic architecture between apoA-IV, kidney function and HDL-cholesterol, while colocalization supports shared causal variants with lipid, renal, and hematological phenotypes, suggesting biologically relevant pathways.

**Conclusion:**

These results provide a comprehensive overview of the genetic architecture of apoA-IV and suggest mechanistic links to lipid metabolism, kidney function and blood-related traits. Our findings refine the role of apoA-IV as a potential biomarker and inform future epidemiological research.

**Supplementary Information:**

The online version contains supplementary material available at https://doi.org/10.1186/s13059-026-04196-7.

## Background

Apolipoprotein A-IV (apoA-IV) is a 46 kDa glycoprotein primarily produced in enterocytes of the small intestine. It is secreted into the lymph as part of chylomicron particles during dietary fat absorption and subsequently enters the bloodstream in both lipoprotein-bound and lipid-free forms [1–3]. ApoA-IV plays a central role in lipid metabolism as a component of high-density lipoproteins (HDL), very-low-density lipoprotein (VLDL), and chylomicrons. Beyond its role in facilitating reverse cholesterol transport, apoA-IV influences satiety [3, 4], insulin secretion [4, 5], and inflammatory response [6, 7], experimental studies have further implicated apoA-IV in chylomicron lipolysis activation [8], cholesterol efflux [9, 10] and inhibition of LDL oxidation [11] and thrombosis [12]. Clinically, low apoA-IV levels have been associated with cardiovascular events [13–16], and elevated apoA-IV has emerged as an early biomarker of impaired kidney function [17–19], highlighting its relevance beyond lipid metabolism.

ApoA-IV is encoded by the *APOA4* gene, located within the *APOA5-A4-C3-A1* cluster on chromosome 11q23 – a region of extensive linkage disequilibrium (LD) among apolipoprotein genes that collectively influence triglyceride and HDL metabolism [20]. While other lipid traits, particularly HDL cholesterol, have been widely studied, apoA-IV has received less attention. In our previous genome-wide association study (GWAS) we identified significant loci for apoA-IV levels in/near *APOA4* and *KLKB1 *[21], but the study was limited to a sample size of 13,813. Recent large-scale proteomics GWAS in more than 33,000 European participants from the UK Biobank (UKB) also identified additional loci (*TDRD5/LAMC1*, *MCUB*, the *HLA* region, *GATA4*, *UMOD* and *APOE*) linked to apoA-IV [22]. However, these findings were embedded in broader multi-protein analyses and lacked detailed investigation of apoA-IV’s genetic regulation.

In addition, little is known about the shared genetic architecture between apoA-IV and traits related to lipids, kidney function, or other blood-based biomarkers, thereby limiting insight into potential pleiotropic or causal mechanisms underlying metabolic and cardiovascular diseases.

To address these gaps, we conducted a GWAS meta-analysis of apoA-IV concentrations in > 25,000 individuals based on ELISA measurements. To further enhance discovery power and evaluate cross-platform robustness, we incorporated summary statistics from an independent proteomics GWAS in 33,995 individuals from the UKB using the Olink platform [22]. We further applied genetic correlation analyses to assess shared genetic architecture, as well as colocalization analyses to identify common causal variants underlying associated signals.

The specific aims of this study were to: 1) to identify genetic loci associated with apoA-IV levels; 2) assess consistency between ELISA- and Olink-based apoA-IV GWAS results; 3) explore genetic correlations between apoA-IV and traits of interest; and 4) pinpoint candidate genes and potential pleiotropic regions via colocalization and conditional association testing. This integrative genomic approach provides new insights into the genetic regulation of apoA-IV and its potential relevance to lipid metabolism, kidney function, and blood-related traits.

## Results

### GWAS on 25,181 individuals based on ELISA-measured apoA-IV concentrations

Genome-wide association analyses were conducted in 25,181 individuals from six population-based studies, one kidney disease cohort, and one diabetes case–control study, with apoA-IV measured by ELISA. Baseline characteristics of each study are presented in Additional file 2: Table S2. ApoA-IV levels were lowest in the youngest cohort, the Young Finns Study (mean = 13.12 mg/dL), and highest in the kidney-disease cohort GCKD (mean = 28.87 mg/dL).

**Fig. 1:**
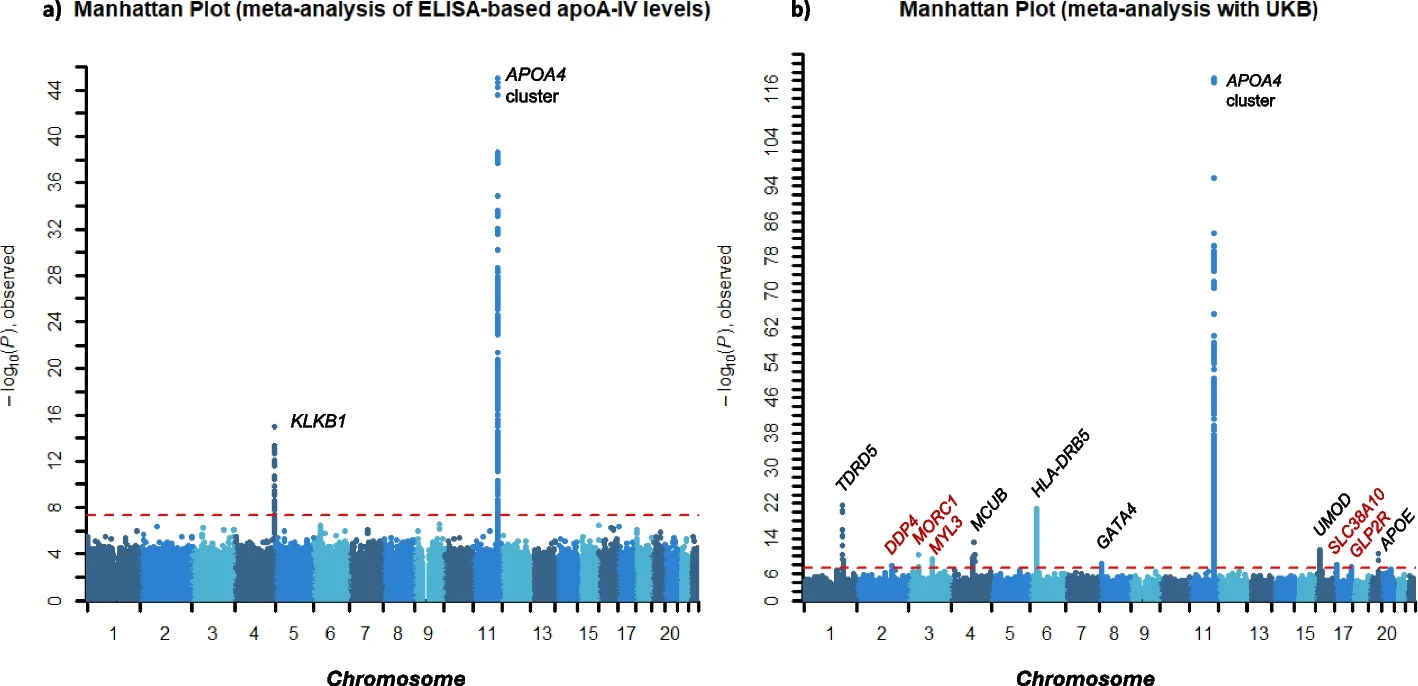
Manhattan plot illustrating genome-wide autosomal associations for apoA-IV. The red line represents the threshold for genome-wide significance (*p*-value < 5 × 10^−08^). The x-axis gives the chromosomes, the y-axis shows the -log10 *p*-values of imputed SNPs. **a** shows the plot for the meta-analysis of all ELISA-based measurements of apoA-IV (*n* = 25,181) adjusted for age, sex, and four principal components (PCs). **b** shows the meta-analysis including the UKB summary statistics (Europeans only) in addition to those shown in plot (**a**) leading to a total of 59,176 individuals. Loci highlighted in red represent signals that reached genome-wide significance only through the increased power of the combined meta-analysis, but not in the UK Biobank dataset alone

The meta-analysis of GWAS on inverse normal-transformed apoA-IV levels (adjusted for age, sex and the first four principal components) identified four independent signals reaching genome-wide significance (*p* < 5 × 10^–08^), shown in the Manhattan plot in Fig. 1a. These included three independent variants in or near the *APOA4* gene and one conditionally independent variant at the *KLKB1* locus (Table 1). The genomic inflation factor for the meta-analysis was λ = 1.013, indicating that population stratification was unlikely to have substantially influenced the results (λ per study ranged from 0.995 to 1.018).

**Table 1:** List of genome-wide significant autosomal lead SNPs from the meta-analysis including ELISA-based apoA-IV data (*n* = 25,181), adjusted for age, sex and the first 4 PCs

Chr:Position	Lead SNP	Ref Allele	Alt Allele	Freq A1 ^a^	Effect ^b^	StdErr	Effect on original scale	Nearest Gene	Distance BP	N	*P*-value	Direction^c^	I^2^	% variance explained
4:187139807	rs12331051	T	C	0.436	−0.070	0.009	−0.26	*KLKB1*	0	25,181	9.87 × 10^–16^	– – – – + – – – + –	78.2	0.24%
11:116684744	rs2849167	A	G	0.511	0.121	0.009	0.53	*APOA4*	6675	25,181	1.00 × 10^–45^	+ + + + + + + + + +	79.2	0.73%
11:116688765	rs9735326	A	C	0.852	0.136	0.012	0.62	*APOA4*	2654	25,181	2.92 × 10^–29^	+ + + + + + + + + +	65.2	0.46%
11:116692293	rs12721043	A	C	0.015	−0.309	0.040	−1.26	*APOA4*	0	21,638	8.37 × 10^–15^	– – –?– – – – – –	54.2	0.27%

^a^Freq A1 = weighted average of frequency for allele 1 across all studies based on our meta-analysis results. ^b^Effect and StdErr based on inverse normal-transformed apoA-IV levels. ^c^order of studies: GCKD, KORA F3, KORA F4, CoLaus, AugUR1, AugUR2, YFS, Diagene, Rotterdam Study 1, Rotterdam Study 2

The strongest association was observed for rs2849167 (β = 0.121, *p* = 1.00 × 10^–45^) and rs9735326 (β = 0.136, *p* = 2.29 × 10^–29^) both located in the *APOA5-A4-C3-A1* gene cluster as well as rs12721043 (β = −0.309, *p* = 8.37 × 10^–15^) in the *APOA4* gene (regional plot shown in Additional file 1: Fig S1), supporting its role in apoA-IV regulation. For all three independent genome-wide significant variants in the *APOA5-A4-C3-A1* cluster, effect directions were mostly consistent across studies, with at most one study per variant showing an opposite effect. On the original scale, these SNPs corresponded to approximate changes in apoA-IV levels of 0.53, 0.62, and −1.26 mg/dL, respectively.

The fourth independent genome-wide significant lead variant, rs12331051, was located in the *KLKB1* region on chromosome 4 (regional plot in Additional file 1: Fig S1, β = −0.07, *p* = 9.87 × 10^–16^), corresponding to a 0.26 mg/dL decrease in circulating apoA-IV levels per effect allele. The *KLKB1* signal showed most significant single-study effect in the GCKD study, whose participants suffer from chronic kidney disease with pronounced elevations of apoA-IV concentrations, still several other cohorts contributed concordant evidence (visualized in a forest plot, Additional file 1: Fig S2). Overall, effect magnitudes were broadly similar across cohorts, albeit with wide confidence intervals in some smaller studies. Study-specific results for all lead SNPs are provided in Additional file 2: Table S3.

Most cohorts collected fasting blood samples, whereas KORA F3, AugUR, and GCKD primarily included nonfasting samples (Additional file 2: Table S2). Stratified meta-analyses comparing fasting and nonfasting cohorts revealed no consistent differences in effect estimates or directions across variants, a finding further supported by visual inspection of forest plots (data not shown), indicating that genetic associations were robust to variation in sample fasting status.

### Sex differences in genetic regulation of apoA-IV

Meta-analyses of sex-stratified GWAS per study were performed to explore potential sex-specific genetic effects. A Z-test comparing effect estimates between men and women identified one variant with significant sex-specific effects (*p*-value of difference = 2.44 × 10^–09^) for the variant rs149287788 near *GSG1L* (effect allele (A)), that showed a negative association with apoA-IV levels in men (β = −0.342), but a positive association in women (β = + 0.242). The minor allele frequency was similar in men and women (0.0086 vs. 0.0087, respectively), and sample sizes were well balanced between sexes (*n* = 10,951 vs. *n* = 10,910, respectively), supporting the robustness of the observed association. Imputation quality was consistently high across all contributing cohorts and study-specific analyses demonstrated consistent effect directions across cohorts (only exception: AugUR2 in men), indicating that the observed sex-specific association is not driven by a single study (visualized in a forest plot, Additional file 1: Fig S3), although replication in larger datasets will be important to further confirm these findings.

Additionally, the *KLKB1* locus reached genome-wide significance only in men (*p* = 3.98 × 10^–08^ vs. *p* = 1.27 × 10^–06^ for the lead SNP at rs12331051), showing a stronger effect in males, however, the *p*-value of difference is not significant (p_diff_ = 0.59) and MAF, effect directions and magnitudes were similar between sexes for this variant (MAF = 0.44, β = −0.065 and standard error 0.0119 in men and MAF = 0.43, β = −0.057 and standard error 0.0117 in women for the top hit at rs12331051).

### Meta-analysis with the UK Biobank summary statistics

To enhance statistical power and evaluate methodological consistency between platforms, we combined our ELISA-based meta-analyzed GWAS results with published GWAS for UKB proteomics data (*n* = 33,995, European ancestry) derived from the Olink platform. The extended meta-analysis included 59,176 individuals (genomic inflation λ = 1.036).

Conditional analysis using summary statistics (GCTA-COJO) identified 18 SNPs independently associated with apoA-IV levels (Table 2***, ***Fig. 1b): The strongest signal originated from the *APOA4* region (lead SNP rs1729407, *p* = 1.43 × 10^–118^), where several conditionally independent SNPs were detected (regional plot shown in Additional file 1: Fig S4). Another strong signal was detected from the *TDRD5* gene (rs150816167, *p* = 2.83 × 10^–22^). Additional conditionally independent variants in or near the following genes were found: *DPP4* (chr 2), *MYL3* and *MORC1* (chr 3), *MCUB* (chr 4), the *HLA-DRA/HLA-DRB1/5* region (chr 6), *GATA4* (chr 8), *ZPR1* (chr 11), *UMOD* (chr 16), G*LP2R/SLC38A10* (chr 17), and *APOE* (chr 19). A comprehensive overview of conditionally independent variants from each study is provided in Additional file 2: Table S4.

**Table 2:** List of conditionally independent, genome-wide significant autosomal lead SNPs from the meta-analysis including ELISA-based and Olink-based apoA-IV data (*n* = 59,176)

Chr:Position	Lead SNP	Ref Allele	Alt Allele	Freq A1^a^	Effect ^b^	StdErr	Nearest Gene	Distance BP	N	meta-analysis *p*-value	Dir ^c^	p_diff_	% variance explained
1:179571862	rs150816167	T	C	0.957	−0.139	0.014	*TDRD5*	0	59,176	2.83 × 10^–22^	– –	0.456	0.16%
2:162964301	rs75166367	A	G	0.062	−0.068	0.012	*DPP4*	33,249	55,633	2.02 × 10^–08^	– –	1	0.05%
3:108865198	rs73216700	A	G	0.426	0.035	0.006	*MORC1*	28,209	59,176	3.96 × 10^–10^	+ +	1	0.06%
3:46889187	rs6787229	A	G	0.102	0.061	0.009	*MYL3*	10,175	59,176	3.45 × 10^–11^	+ +	1	0.07%
4:110578226	rs189866430	A	T	0.007	−0.281	0.037	*MCUB*	0	57,073	5.48 × 10^–14^	– –	1	0.11%
6:32429582	rs9268851	A	G	0.139	−0.080	0.008	*HLA-DRB5*	55,538	54,830	1.15 × 10^–21^	– –	1	0.15%
6:32503930	rs34582150	T	C	0.846	0.081	0.009	*HLA-DRB5*	5866	42,679	4.39 × 10^–18^	+ +	1	0.17%
6:32574575	rs113315602*	A	C	0.913	−0.018	0.012	*HLA-DRB1*	16,950	52,727	0.141	+ –	1	0.01%
8:11530493	rs10903342	A	C	0.533	−0.031	0.006	*GATA4*	31,220	59,176	2.95 × 10^–08^	– –	0.026	0.05%
11:116646859	rs145648042	A	G	0.002	−0.399	0.070	*ZPR1*	1577	50,217	1.12 × 10^–08^	– –	1	0.07%
11:116677370	rs1729407	C	G	0.469	−0.132	0.006	*APOA4*	14,049	59,176	1.43 × 10^–118^	– –	1	0.86%
11:116691634	rs5110*	A	C	0.085	−0.032	0.011	*APOA4*	0	59,176	0.0021	+ –	**3.76 × 10**^**–09**^	0.02%
11:116692293	rs12721043	A	C	0.012	−0.169	0.027	*APOA4*	0	55,633	5.39 × 10^–10^	– –	**2.50 × 10**^**–05**^	0.07%
11:116692334	rs5104	T	C	0.866	0.138	0.008	*APOA4*	0	59,176	5.95 × 10^–61^	+ +	1	0.44%
16:20361950	rs36060036	T	C	0.165	−0.052	0.008	*UMOD*	0	59,176	5.88 × 10^–12^	– –	1	0.07%
17:79218714	rs2659005	T	C	0.448	0.032	0.006	*SLC38A10*	87	56,272	2.87 × 10^–08^	+ +	1	0.05%
17:9792768	rs17681684	A	G	0.315	0.035	0.006	*GLP2R*	0	55,633	1.10 × 10^–08^	+ +	1	0.05%
19:45412079	rs7412	T	C	0.082	0.071	0.011	*APOE*	0	55,633	2.03 × 10^–11^	+ +	1	0.08%

^a^Freq A1 = weighted average frequency for allele 1 across all studies based on our meta-analysis results. ^b^Effect and StdErr based on inverse normal-transformed apoA-IV levels. ^c^order of studies: ELISA meta-analysis, UKB summary statistics. SNPs marked * were not genome-wide significant in the meta-analysis (*p*-value of meta-analysis ≥ 5 × 10^–08^) but became significant after conditioning on other independent variants (*p*-value of conditional analysis < 5 × 10^–08^). The platform-specific effect differences (p_diff_) were calculated using a z-test comparing the effect estimates in UKB data and ELISA-based data$({\beta}_{UKB}-{\beta}_{ELISA})/\sqrt{Std{Err}_{UKB}^{2}+S{tdErr}_{ELISA}^{2}}$, and the resulting *p*-values were evaluated against a Bonferroni-corrected significance threshold of 0.00278 (0.05/18)

Inspection of the forest plots revealed that almost all studies showed effects in the same direction (see exemplary forest plots for lead SNPs rs2849167 for the ELISA-based meta-analysis and rs1729407 for the combined meta-analysis in Additional file 1: Fig S5).

When evaluating the 18 variants identified in the extended meta-analysis for difference in platform-specific effect estimates (ELISA-based studies meta-analysis versus OLINK in UKB), we found no evidence for difference, except for two variants near *APOA4* (rs5110 and rs12721043 with p_diff_ < 0.05/18 (3.76 × 10^–09^ and 2.50 × 10^–05^, respectively). rs5110 was a variant that was not genome-wide significant in the meta-analysis (*p*-value of meta-analysis ≥ 5 × 10^–08^) but became significant after conditioning on other independent variants (*p*-value of conditional analysis < 5 × 10^–08^).

Notably, neither the index variant in *KLKB1* nor another variant in this locus did not reach genome-wide significance in this extended analysis (*p*-value: 4.80 × 10^–07^ for rs12331051). This attenuation is driven by the absence of an effect in the UKB dataset (β ± SE: −0.07 ± 0.009 in the ELISA-based studies vs. 0.003 ± 0.008 in the UKB, respectively, with a p_diff_ of 1.34 × 10^–09^), as illustrated in Additional file 1: Fig S2. Consequently, this association observed in the ELISA-based analysis does not persist when UKB data are included (see Discussion).While the *KLKB1* locus reached genome-wide significance only in the ELISA-based apoA-IV GWAS, other loci (e.g., *DPP4*, *MYL3*, *MORC1*, *GLP2R* and *SLC38A10*) reached genome-wide significance only in the combined analysis, highlighting the gain in statistical power.

### Impact of kidney function and diabetes adjustment on lead SNPs

Adjustment for kidney function parameters was performed across all studies, using individual-level data where available. In the ELISA-based studies, adjustment for eGFR was feasible in 25,139 out of 25,181 individuals and had minimal impact on the effect sizes and *p*-values of the four conditionally independent SNPs identified. No additional locus reached genome-wide significance following adjustment.

In addition, an approximate conditional analysis adjusting for either eGFR or UACR was conducted based on the total meta-analysis summary statistics (*n* = 59,176), which had no major effects on the main findings. Effect estimates for all independent lead SNPs remained stable and directionally consistent after accounting for kidney function. Notably, at the *UMOD* locus (lead SNP rs36060036), the *p*-value changed from 5.88 × 10^–12^ to 4.77 × 10^–09^ after eGFR-adjustment, while the effect size remained stable (β = −0.052 adjusted for age and sex; β = −0.048 after additional adjustment for eGFR; and β = −0.053 after adjustment for UACR), indicating that only a small part of the association of the *UMOD* variant with apoA-IV levels is mediated by kidney function defined by eGFR or UACR. Similarly, additional adjustment for type 2 diabetes resulted in only modest attenuation or slight strengthening of selected associations, with no changes in effect direction and no variants losing genome-wide significance. Adjusted beta estimates and *p*-values are presented in Additional file 2: Table S5.

To further assess whether observed associations were disproportionately driven by disease-enriched cohorts (GCKD, a chronic kidney disease cohort; DiaGene, a diabetes case–control study), leave-one-cohort-out meta-analyses were performed for all lead variants. Effect directions remained consistent, and effect sizes and *p*-values did not materially change upon the exclusion of any single cohort from the combined meta-analysis including UK Biobank (Additional file 2: Table S6). Heterogeneity metrics (Cochran’s Q and I^2^) for the top loci, together with pooled estimates omitting one cohort at a time, are also provided in Additional file 2: Table S6. In ELISA-based analyses only, attenuation was observed for two variants upon exclusion of GCKD (rs12331051 at *KLKB1* and rs12721043 at *APOA4*), reflecting the larger contribution of this cohort to the ELISA-only meta-analysis.

### Cross-platform concordance of genetic associations

**Fig. 2:**
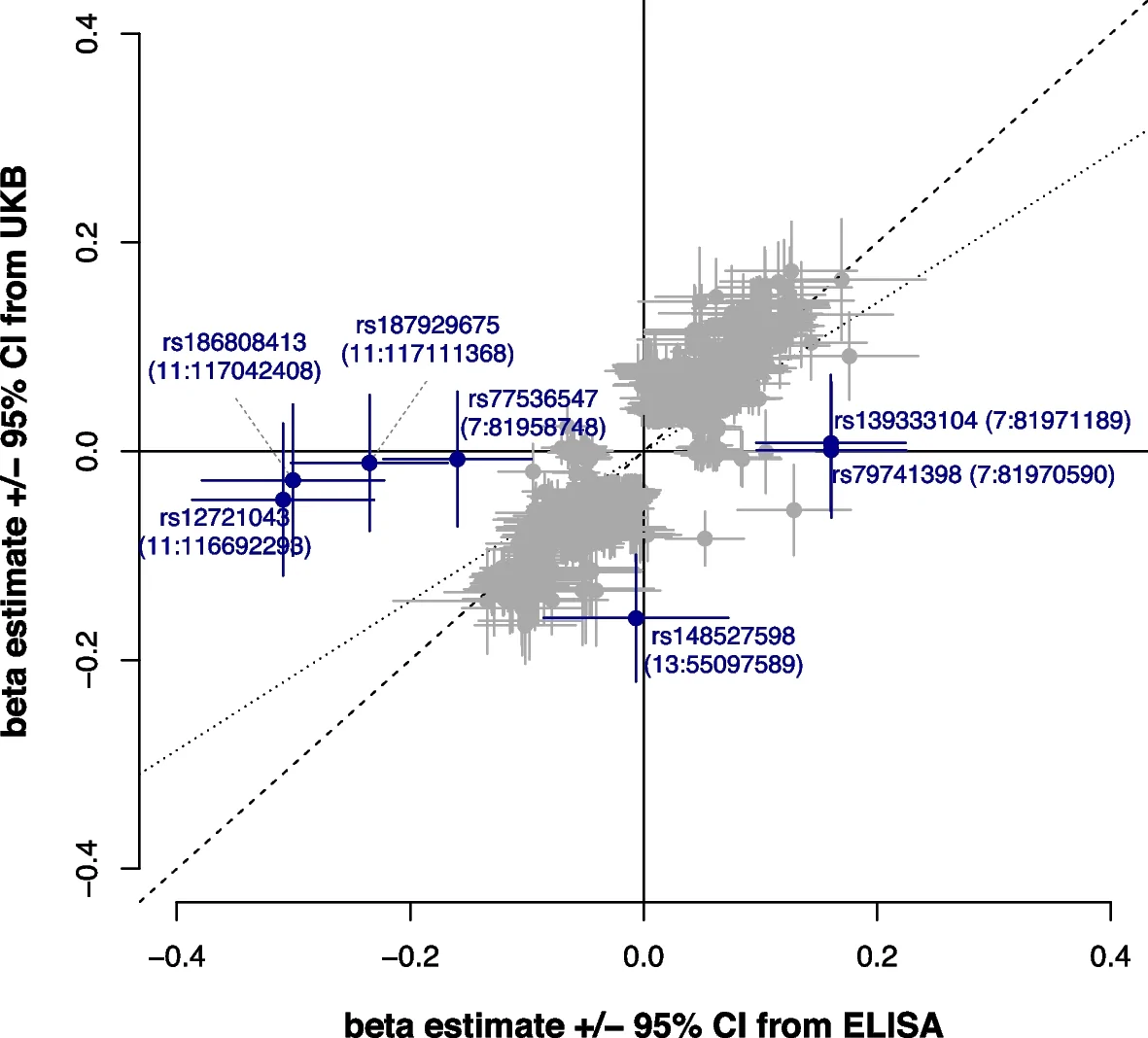
Scatterplot of effect size (β) comparison between ELISA and Olink Assay based on GWAS results on inverse normal-transformed apoA-IV levels. Each point represents a SNP that is shared between both datasets and had a *p*-value < 1 × 10^–06^ in at least one of the datasets. The x-axis shows the β estimates from the ELISA-based analysis, while the y-axis displays the β estimates from the Olink-based analysis. The dashed diagonal line (black) represents the expected trend if the effect sizes were identical between the two methods. SNPs not meeting the equivalence criterion (equivalence margin of 0.33 × the phenotypic variance) are highlighted in blue. When applying a more stringent equivalence margin (Δ = 0.1), more than 80% of variants remain significantly equivalent; variants not meeting this stricter criterion are indicated in dark grey. The dashed regression line represents the best-fit linear regression model between the two datasets, capturing the overall trend of the β-comparison

To assess the concordance between the GWAS results based on ELISA-measured apoA-IV and those from the UKB Olink proteomics platform, we evaluated both the overall correlation and consistency of effects for shared variants with *p* < 1 × 10^–06^ in at least one dataset. The Pearson correlation coefficient (*r* = 0.88, *p* < 2.2 × 10^–16^) further confirmed a strong linear relationship between effect sizes from the two summary statistics, and 97% of SNPs showed concordant directions of effect. Using equivalence testing with a predefined margin of Δ = 0.33, more than 99% of the 2,110 shared SNPs were classified as statistically equivalent. The remaining seven variants did not meet the equivalence criterion and correspond to those showing the most pronounced cross-platform differences (visualized in Fig. 2, with these seven variants highlighted by color). Application of a more stringent margin (Δ = 0.1) reduced the proportion of equivalent variants to 83%, while still indicating substantial overall agreement between platforms. These results suggest that the two measurement platforms yield highly comparable genetic associations for apoA-IV levels, with differences only in a limited number of variants.

### Functional annotation and database lookup: loci associated with diverse biological pathways

In addition to the lead signal at the *APOA4* locus, several variants identified in our GWAS have been previously associated with other phenotypic traits. According to the Common Metabolic Diseases Knowledge Portal (CMDKP, https://hugeamp.org/ accessed March 13, 2025), many lead variants showed genome-wide associations with lipid traits (e.g., HDL-C, LDL-C, triglycerides), kidney function (e.g., eGFR, cystatin C), and inflammatory or hematological parameters (e.g., C-reactive protein, blood cell traits). Notable overlaps included lipid-related signals near *APOE, MCUB, MORC1, MYL3,* and *GLP2R*; kidney-related associations at *UMOD, DPP4,* and *GATA4*; and immune-related signals near *DPP4, HLA-DRB5,* and *GATA4*. These findings informed the selection of phenotypes for downstream genetic correlation and colocalization analyses.

Gene-based testing with MAGMA identified several significant genes beyond the well-known and already highlighted *APOA5-A4-C3-A1* cluster, the *HLA* region, and *UMOD*, including *SIK3, PAFAH1B2, MTTP,* and *MSRA* (Additional file 1: Fig S6). Additional file 2: Table S7 lists all 45 protein-coding genes (out of the 20,043 tested) that reached genome-wide significance based on the MAGMA gene-based test (*P* < 2495 × 10^–06^), with genes newly identified by the gene-based analysis (i.e., not previously highlighted in the SNP-based meta-analysis) shown in bold. A total of 18 lead SNPs were identified via GCTA-COJO conditional analysis, compared to 34 lead SNPs from FUMA’s LD-based clumping. The smaller number of signals from GCTA-COJO reflects its more stringent definition of independence based on conditional modeling, in contrast to the pairwise LD-based approach applied in FUMA. Of these 34 SNPs (shown in Additional file 2: Table S8), around 57% are located within intronic regions, followed by intergenic (26.3%) and untranslated or regulatory regions (Additional file 1: Fig S7).

A gene expression heatmap is provided in Additional file 1: Fig S8, showing log₂-transformed average expression levels across tissues, with genes ordered by hierarchical clustering. Tissue-specific expression analysis using the GENE2FUNC module in FUMA (GTEx v8, 30 general tissue types) revealed enrichment of the prioritized genes among upregulated genes (especially in liver, and kidney), suggesting tissue-specific functional relevance. However, these enrichments did not remain significant after correction for multiple testing.

Pathway enrichment analysis using Reactome showed significant overrepresentation of genes involved in lipoprotein metabolism, including plasma lipoprotein assembly, clearance, and remodeling, largely driven by *APOA4, APOA1, APOC3, APOE*, and *MTTP*. GWAS Catalog enrichment revealed substantial overlap with loci previously associated with lipid traits, as well as cardiometabolic and neurological phenotypes, including Alzheimer’s disease and cognitive function, again predominantly involving apolipoprotein genes (*APOA1, APOA4, APOC3, APOC1,* and *APOE*). Further details are provided in Additional file 1: Fig S6, showing results of gene-based analyses and functional annotations.

### Global genetic correlation

**Fig. 3:**
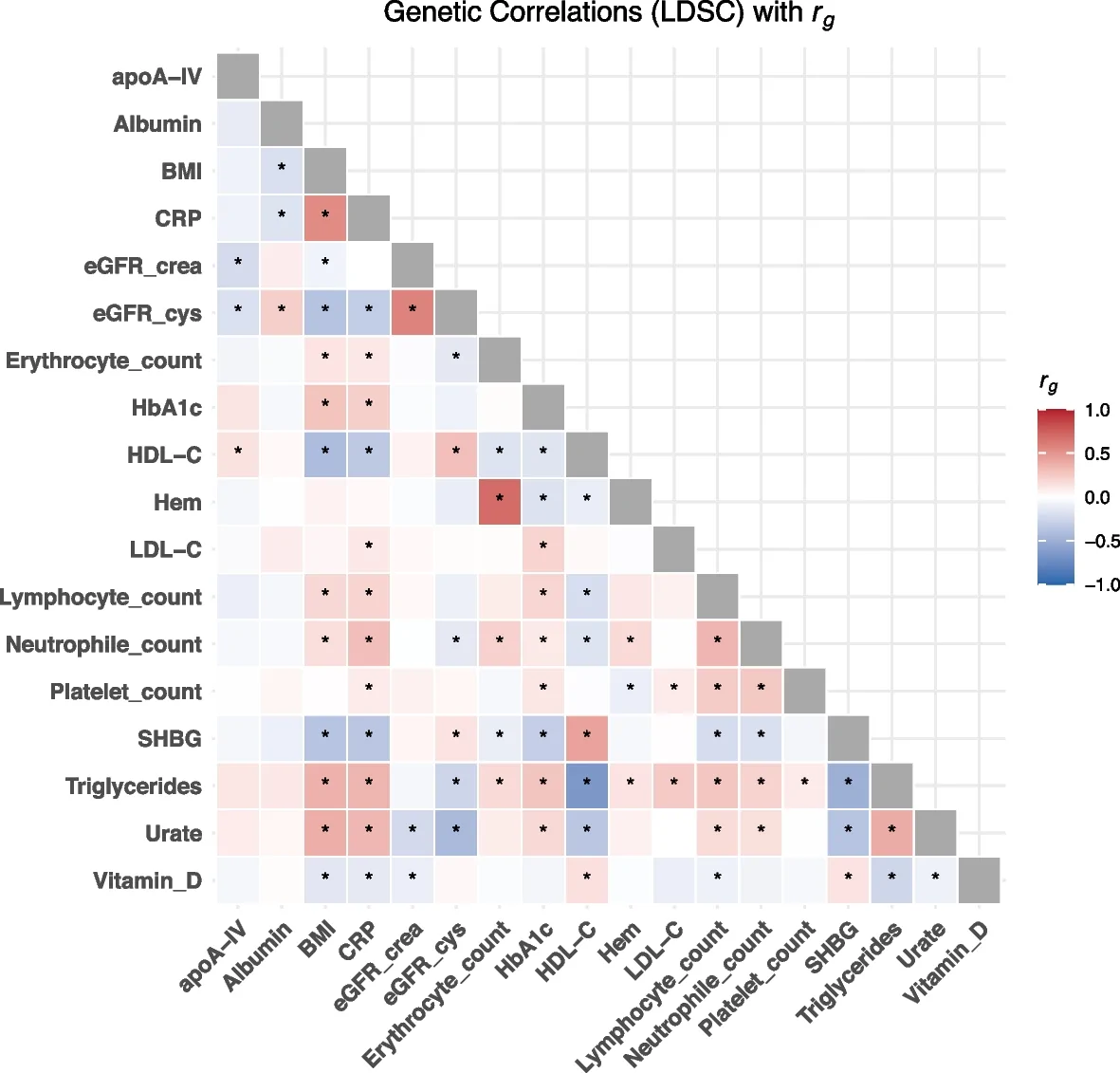
Global genetic correlation between apoA-IV and traits of interest according to LDSC. Significant genetic correlations after Holm-Bonferroni correction for multiple testing (adjusted *p* < 0.05) are highlighted with asterisks. Abbreviations: Hem = hemoglobin; SHBG = sex hormone-binding globulin

The LDSC approach indicated that apoA-IV shares an overall genetic architecture with eGFR based on cystatin C (eGFR_cys_), eGFR based on creatinine (eGFR_crea_), and HDL-C. After Holm-Bonferroni correction for multiple testing, all three traits showed significant genome-wide genetic correlations with apoA-IV (adjusted *p* = 0.001, 0.035, and 0.046, respectively). The correlations were positive for HDL-C and inverse for both eGFR measures, as expected. The observed genetic correlations represented the strongest associations among all traits examined and showed small-to-moderate magnitudes (r_g_ = − 0.22, − 0.19, and 0.14 for eGFRcrea, eGFRcys, and HDL-C, respectively). The full correlation matrix displaying genome-wide genetic correlations r_g_ is shown in Fig. 3.

### Colocalization analysis

**Fig. 4:**
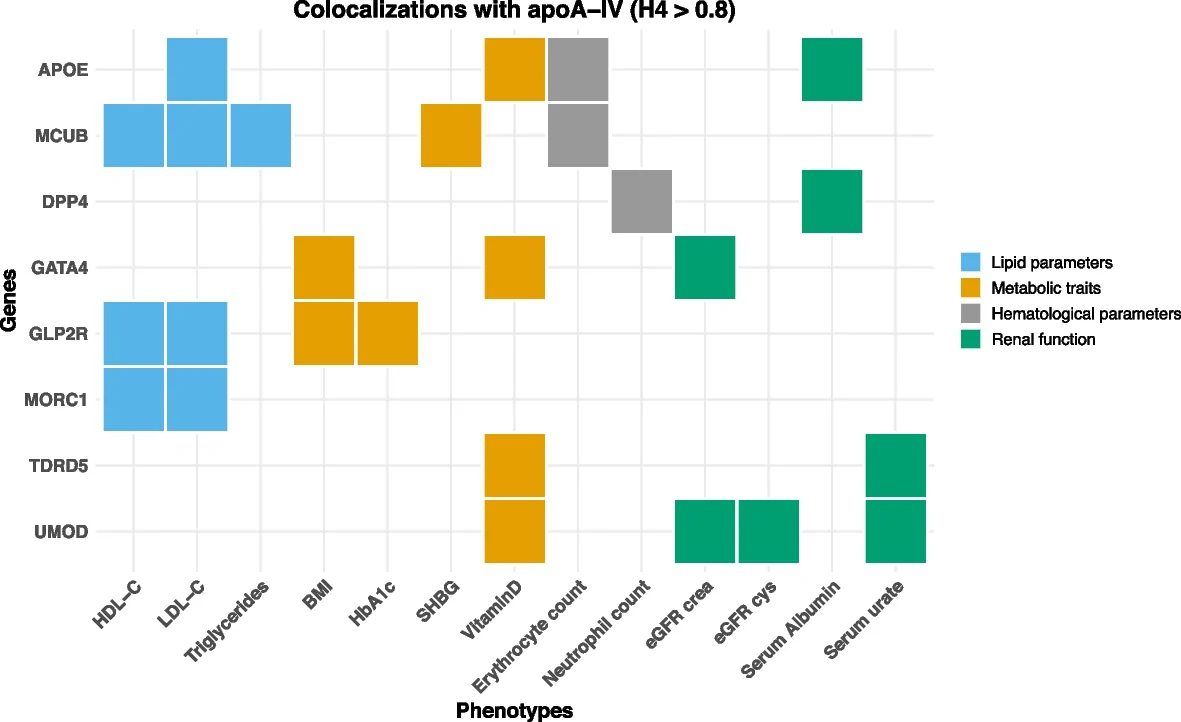
Positive colocalization signals between apoA-IV-associated loci and other phenotypes, depicting phenotypes with strong colocalization signals (posterior probability for shared causal variant, H4 > 0.8) at genes identified in the apoA-IV GWAS

Using data from the same phenotypes and traits of interest, colocalization analyses (using marginal summary statistics) revealed evidence of shared genetic signals (H4 > 0.8) between apoA-IV levels and 13 phenotypes across eight genes (Fig. 4). Most notably, lipid-related traits (HDL-C, LDL-C, and triglycerides) colocalized with apoA-IV at four loci: *APOE, MCUB, GLP2R*, and *MORC1*, supporting the known involvement of apoA-IV in lipid metabolism. In addition, several colocalizations were observed with kidney function traits, particularly at the *UMOD* and *TDRD5* loci (eGFR_crea_, eGFR_cys_, and serum urate). Colocalizations with metabolic traits were also observed at multiple genes. BMI and HbA_1c_ colocalized at *GLP2R* and *GATA4*, and sex-hormone binding globulin (SHBG) colocalized at *GATA4* and *MCUB*. Additionally, vitamin D colocalized at *GATA4, TDRD5*, and *APOE*, suggesting potential links between apoA-IV and broader endocrine or metabolic regulation. Additionally, hematological parameters such as neutrophil and erythrocyte counts showed colocalizations with *DPP4, MCUB* and *APOE*. No colocalization signal was detected for the *APOA4* cluster, although SNPs within that cluster are also associated primarily with HDL-C. These SNPs are not in LD with the apoA-IV top hits though, indicating distinct genetic architectures. Exemplary colocalization plots illustrating strong pleiotropy at the *APOE* locus and no evidence for main signal colocalization at the *APOA4* locus are shown in Additional file 1: Fig S9.

Together, these findings underscore the pleiotropic nature of apoA-IV and suggest that shared genetic architecture underlies its involvement in lipid metabolism, metabolic traits, kidney function, and selected hematological/metabolic parameters.

## Discussion

In this large-scale GWAS of ELISA-measured apoA-IV levels, we identified four genome-wide significant loci, including three within the *APOA5-A4-C3-A1* gene cluster and one at the *KLKB1* locus. Meta-analysis including UK Biobank Olink data extended these findings to 18 conditionally independent signals, highlighting several novel loci involved in lipid metabolism, kidney function, and inflammation. Strong cross-platform consistency and genetic correlations with traits such as HDL-C and eGFR support the robustness and biological relevance of these associations. Colocalization analyses revealed widespread shared genetic architecture with a broad spectrum of traits, consistent with systemic pathways influencing apoA-IV as a potential downstream marker. However, the absence of colocalization at the *APOA4* locus itself suggests that this signal likely reflects a locus-specific, primary effect on apoA-IV regulation rather than secondary consequences of polygenic, system-wide metabolic differences. Together, these findings provide new insights into the genetic regulation of apoA-IV and lay the foundation for understanding its broader physiological relevance in human health and disease.

Although apoA-IV is increasingly recognized for its regulatory roles in lipid metabolism, satiety, and glucose homeostasis, much of the current evidence is based on animal models or small-scale studies conducted decades ago [3, 4, 6, 23]. Large-scale human data remain limited and are often derived from disease-specific cohorts, such as individuals with chronic kidney disease, thereby restricting broader applicability [19, 24, 25]. The strongest evidence supports a role for apoA-IV in kidney disease across different stages, with higher apoA-IV levels consistently associated with reduced kidney function [17, 18, 24, 26, 27].

To date, only one GWAS has investigated apoA-IV concentrations measured via ELISA, conducted previously by our group and involving overlapping study populations [21]. In that GWAS, five population-based cohorts (*n* = 13,813) and two replication cohorts (*n* = 2,267) were analyzed using 1000 Genomes imputation reference panel with the following conditionally independent lead SNPs: rs1729407 (near *APOA4*, *p* = 6.77 × 10^−44^), rs5104 (in *APOA4*, *p* = 1.79 × 10^−24^) and rs4241819 in the *KLKB1* gene (*p* = 5.6 × 10^−14^), respectively [21]. A follow-up Mendelian randomization study based on this GWAS suggested potential causal links between apoA-IV and HDL-C, and potential reverse causality between apoA-IV and both, eGFR and triglycerides [28]. However, disentangling the contribution of HDL-C and LDL-C to apoA-IV from that of triglycerides posed a challenge [28].

### Platform-specific effects and interpretation of complex loci

With the availability of UK Biobank proteomic data (including almost 3,000 different proteins) from > 30,000 European UKB participants measured via the Olink platform, plasma proteomic GWAS have expanded considerably [22]. Although several additional loci, such as *NPHS2*, *LAMC1*, and *C3orf85*, achieved genome-wide significance in the GWAS on apoA-IV by Sun et al., their effect sizes were relatively small, further emphasizing *APOA4* as the primary genetic regulator of apoA-IV [22]. The strong concordance between ELISA- and Olink-based GWAS results for apoA-IV underscores the robustness of these genetic associations. Despite discrepancies in a few variants, both approaches revealed largely consistent effect directions and magnitudes, supporting the reliability of cross-platform findings.

Building on this concordance, combining results from both platforms in a meta-analysis appears justified. Although ELISA and Olink differ in assay chemistry, the inverse normal-transformation applied across cohorts and the overall consistency of genetic effect estimates indicate that both approaches capture a shared underlying genetic signal. Sensitivity analyses using random-effects models produced highly similar results (Additional file 2: Table S4). As expected, a small number of variants with substantial between-study heterogeneity and stronger effects in the large UKB cohort showed attenuated effect estimates and lost significance under the random-effects model, reflecting the reduced influence of the largest study compared with the fixed-effect analysis. This likely reflects both the accommodation of between-study heterogeneity and the reduced relative influence of the UK Biobank cohort when individual ELISA studies were modeled separately rather than combined prior to meta-analysis.Collectively, these findings support the use of a fixed-effect model for the primary meta-analysis while acknowledging inherent technical differences between measurement platforms.

One notable difference emerged at the *KLKB1* locus. This locus reached a clear genome-wide significance in our ELISA-based meta-analysis, consistent with earlier findings [21], but did not achieve genome-wide significance in the Olink-based UK Biobank dataset [22]. Several factors may contribute to this discrepancy, including methodological differences such as antibody specificity, sample handling, and fasting status, as well as biological variation arising from the coexistence of multiple molecular forms of apoA-IV. Moreover, *KLKB1* encodes plasma kallikrein, a calcium-sensitive protease involved in the processing and modification of plasma proteins, making it plausible that *KLKB1* variants alter structural or post-translational features of apoA-IV and thereby affect its detectability in a platform-dependent manner. Importantly, we speculate that such effects might be limited to loci like *KLKB1* that modify apoA-IV’s biochemical state, and not to loci that mainly influence total apoA-IV abundance (e.g., *APOA4*), which may explain why the discrepancy is observed only at *KLKB1*. Whether specific modified forms of apoA-IV and other structural alterations might be less reliably detected via the Olink technology, remains to be determined. Ultimately, given the limited information available on the Olink antibodies and the absence of direct comparisons of identical samples across platforms, the underlying cause of this discrepancy cannot be conclusively determined. Thus, the complete absence of genome-wide significance for the *KLKB1* locus in the UK Biobank likely reflects both technical and biological differences, underlining the importance of contextual interpretation in cross-platform genetic studies. Still, identified discrepancies in the *KLKB1* locus require further investigation.

Moreover, several variants within the *HLA* region reached genome-wide significance in our combined meta-analysis. However, this region is highly polymorphic, exhibits complex linkage disequilibrium, and presents known challenges for genotyping accuracy [29, 30]. These signals were driven almost exclusively by the UKB dataset with very broad confidence intervals in most ELISA-based studies, none reached nominal significance (*p* < 2.1 × 10^–03^) in the ELISA-based cohorts. Given the known issues of genotyping and imputation in this region, as well as its structural variability, these associations should be interpreted with caution [29, 30]. Biologically, the signal may suggest an immune-metabolic link involving apoA-IV, but independent replication is required to draw firm conclusions.

Finally, the observed sensitivity of the *UMOD* locus to kidney function adjustment provides important insights into the relationship between renal physiology and apoA-IV regulation. The top signal at rs36060036 in *UMOD* identified in our apoA-IV meta-GWAS is in strong linkage disequilibrium with rs12917707, a well-characterized uromodulin variant, suggesting a shared genetic basis, which is further supported by colocalization analysis. Uromodulin, encoded by *UMOD*, is the most abundant protein in normal urine and is secreted exclusively by cells in the thick ascending limb of the loop of Henle, highlighting its role for tubular function. It is involved in salt handling, and urinary tract defense, with genetic variants at this locus strongly associated with chronic kidney disease and kidney biomarkers [31]. Although the *UMOD* association signal was slightly attenuated after adjustment for eGFR, it remained genome-wide significant, suggesting that kidney function partly mediates this association, yet an independent effect on apoA-IV beyond filtration function is evident. These findings emphasize the importance of adjusting for renal parameters in apoA-IV-related analyses.

### Broader functional relevance and pleiotropic genetic links of apoA-IV-associated loci

The cross-trait overlap observed in our GWAS highlights a multifaceted role for apoA-IV, extending beyond lipid transport. Several loci, including those near *APOE*, *MCUB*, *MORC1*, and *MYL3*, were associated with lipid traits, aligning with apoA-IV’s established function in lipid metabolism. For example, *GATA4* variants are associated with triglyceride levels in humans, and experimental studies in mice show that hepatic GATA4 regulates triglyceride homeostasis [32–34]. Associations near *UMOD*, *DPP4*, and *GATA4* with renal markers (e.g., eGFR, creatinine, cystatin C) support previous findings of an inverse relationship between apoA-IV and eGFR [17, 19]. Importantly, in an earlier study from our group, we further reported that in individuals with nephrotic syndrome, but not in others, serum apoA-IV correlates positively with serum albumin. This suggests that impaired tubular reabsorption increases urinary apoA-IV excretion, thereby lowering circulating apoA-IV levels compared to individuals without pronounced tubular dysfunction [27].

Additional loci, notably *DPP4*, *GATA4*, and *HLA-DRB5*, were associated with immune and inflammatory phenotypes, including blood cell traits and C-reactive protein levels. The significant associations within the *HLA* region, though driven predominantly by the UKB data, raise the possibility of immune-metabolic crosstalk involving apoA-IV. The association of *GATA4* with both lipid and hematological traits further supports a broader systemic role.

Gene-based and functional annotation analyses further supported these pleiotropic links, with significant enrichment in liver and kidney tissues, emphasizing the organ-specific relevance of apoA-IV-associated loci. We observed enrichment of genes related to lipid traits and metabolism, with overlaps involving cardiovascular risk factors, kidney function, and neurological traits such as Alzheimer’s disease. Together, these findings point to broader systemic roles for apoA-IV, potentially bridging metabolic and neurodegenerative pathways.

To the best of our knowledge, this study represents the largest GWAS on apoA-IV concentrations to date. All ELISA-based studies were processed using a fully harmonized workflow across phenotype curation, genotyping, and analysis, and employed a standardized GWAS pipeline (nf-gwas [35]), ensuring maximum comparability and methodological consistency. In this project, apoA-IV levels were inverse normal-transformed to enhance comparability. This project incorporated eight well-characterized cohorts, mostly population-based, with careful consideration of heterogeneity. Although apoA-IV concentrations are known to be influenced by fasting status, our stratified analyses showed no systematic differences in genetic effect estimates between fasting and nonfasting cohorts. This observation is consistent with recent reports suggesting that fasting status has minimal impact on protein quantitative trait locus discovery and supports the robustness of our findings across heterogeneous sampling conditions [22].

Limitations of this study include the European ancestry of participants, which may limit the generalizability of the findings to other ethnic groups. The participating cohorts comprised mainly population-based studies but also included a chronic kidney disease cohort (GCKD) and case–control study of individuals with and without diabetes (DiaGene), which may contribute to heterogeneity in phenotype distributions. However, leave-one-cohort-out analyses indicated that the observed associations were not disproportionately driven by any single study. Moreover, apoA-IV exists in multiple molecular forms (lipid-bound, lipid-free, or post-translationally or structurally modified) which may be differentially detected by ELISA versus Olink technology. Robust fine-mapping was limited by the meta-analytic design and the lack of cohort-specific LD information, and definitive causal inference would require harmonized individual-level data or study-specific LD matrices. Finally, although Mendelian randomization (MR) represents a potential approach to assess causal relationships with renal and cardiometabolic traits, several methodological challenges (including limited suitable cis-instruments, extensive pleiotropy, and partial sample overlap with large outcome GWAS) currently complicate robust causal inference. A comprehensive MR framework would therefore be better addressed in a dedicated follow-up study.

## Conclusions

Our cross-platform GWAS meta-analysis significantly advances the understanding of the apoA-IV genetics by identifying multiple robust and novel loci associated with lipid metabolism, kidney function, and systemic traits. By integrating ELISA measurements with Olink proteomic data from the UK Biobank in over 59,000 individuals, we show strong concordance of genetic effects across platforms, supporting the reliability and biological validity of apoA-IV quantification. This methodological comparison highlights the importance of cross-platform validation in an era of large-scale proteomics and highlights apoA-IV’s potential as both a biomarker and a potential mediator of cardiometabolic disease. Future studies should focus on functional validation of identified key loci and explore clinical implications of apoA-IV across diverse populations.

## Methods

### Study populations

All studies were carried out in accordance with approved guidelines and in compliance with the Declaration of Helsinki and relevant national and EU regulations. Written informed consent was obtained from all participants in all included studies, and all studies possess ethical approval from local or institutional ethics committees. Detailed study information is provided in the Supplemental Materials (Additional file 1). There is no known sample overlap between any of the cohorts included in this meta-analysis.

### Measurements of apolipoprotein A-IV

ApoA-IV was measured in serum or plasma via ELISA in eight cohorts (KORA F3, KORA F4 [36], AugUR [37, 38], the GCKD study [39, 40], CoLaus [41], Young Finns Study [42], DiaGene [43], Rotterdam [44, 45]) at the Institute of Genetic Epidemiology, Innsbruck, Austria, using a standardized protocol [17, 46]. Briefly, a double-antibody enzyme-linked immunosorbent assay (ELISA) was applied, using an affinity-purified polyclonal rabbit anti-human apoA-IV antibody for both coating and detection (the latter conjugated to horseradish peroxidase). Calibration was based on a standard with known apoA-IV concentrations, and each assay included four internal controls across concentration ranges to ensure consistency across studies [46]. Quality control procedures included close monitoring of intra-assay and inter-assay variability to maintain high measurement precision.

### Genome-wide association study and GWAS meta-analysis

Cohort-specific genotyping, imputation, and quality control (QC) procedures are summarized in Additional file 2: Table S1. All studies were imputed using the Haplotype Reference Consortium (HRC) reference panel. Variants with low imputation quality scores (INFO score < 0.3) were excluded.

GWAS were performed separately in each cohort using the REGENIE-based Nextflow pipeline nf-gwas (available at https://genepi.github.io/nf-gwas/) [35]. Genotype data were quality controlled with standard steps (using default parameters unlessstated otherwise) in the Nextflow pipeline before the association analysis with REGENIE [47]. The REGENIE algorithm involves two steps: (1) a whole-genome regression model to account for population structure and relatedness, using LD-pruned genotyped variants (window size: 1000 variants, step size: 100 variants, *r*^2^ < 0.9) and (2) single-variant association testing conditioned on predictions from step 1 [47]. Analyses were based on HRC-imputed dosages using linear regression models in an additive model. ApoA-IV concentrations were inverse normal-transformed to enable comparability across cohorts, and β-estimates of lead SNPs were also reported on the original scale. Covariates included age, sex, and the first four principal components (PCs). Extended models were further adjusted for estimated glomerular filtration rate (eGFR) and urinary albumin-to-creatinine ratio (UACR), due to the established relationship between kidney function and apoA-IV levels [17]. Sex-stratified GWASs were performed to account for known sex differences in apoA-IV concentrations [17].

All ELISA-based studies followed a harmonized workflow using the same pipeline and parameter settings. Statistical significance for variant-level (GWAS) associations was defined as *p* < 5 × 10^−08^. Phenotype data processing and post-GWAS analyses were conducted in R version 4.4.1 (R Foundation for Statistical Computing, Vienna, Austria), unless otherwise stated. Meta-analysis of summary statistics from the ELISA-based cohorts (*n* = 25,181) was conducted using METAL [48] (fixed-effect inverse-variance weighted) with heterogeneity assessed via I^2^ statistics. Within each locus, the most significant variant (top hit; defined by lowest *p*-value) was selected as the initial input for conditional analyses. Conditional analyses were conducted using GCTA-COJO [49] within ± 250 kb regions (*p*-value cutoff = 5 × 10^–08^; collinearity cutoff = 0.9), with the GCKD cohort as reference for the LD structure. Variants that showed conditionally independent associations with apoA-IV concentrations were considered lead SNPs. To account for potential between-study heterogeneity, we re-evaluated lead SNPs with high heterogeneity in the fixed-effect meta-analysis (I^2^ > 50%) using a random-effects model implemented in the metafor R package (method = REML). For each conditionally independent lead SNP, the proportion of variance explained was estimated as r^2^ = β^2^/(β^2^ + n × SE^2^), where β is the effect estimate, SE its standard error, and n the sample size. To assess the robustness of association signals to cohort composition, leave-one-cohort-out meta-analyses were conducted by sequentially excluding each cohort and re-running inverse-variance weighted meta-analysis for all lead variants. Between-study heterogeneity was summarized using Cochran’s Q statistic and I^2^.

### UK Biobank GWAS

To increase discovery power and enable cross-platform comparison, we included summary statistics from the publicly available UK Biobank proteomics GWAS (https://www.synapse.org/Synapse:syn52361547). Genotyping, imputation, and quality control for UK Biobank data have been described previously [50], and details specific to the proteomics GWAS using the Olink platform were reported by Sun et al. [22] Briefly, GWAS was conducted using REGENIE v2.2.1 across > 2,900 proteins measured by proximity extension assay (PEA). Step one used high-quality genotyped variants (MAF > 1%, MAC > 100, call rate > 99%, HWE *p* > 10^–15^), followed by linear regression (step two) with leave-one-chromosome-out predictions. Variants were further filtered by INFO score > 0.7 and MAC > 50 to reduce false signals. Protein levels were inverse-rank normalized (including values below the detection limit), and association models were adjusted for age, sex, sampling batch, genotyping array, blood sampling time, and the first 20 PCs [22, 50]. Our study included only UK Biobank participants of European ancestry (*n* = 33,995).

### Meta-GWAS of ELISA- and Olink-derived apoA-IV data

In addition to our ELISA-based meta-GWAS (*n* = 25,181), we performed an extended meta-analysis using METAL [48] (fixed-effect inverse-variance weighted) incorporating the UKB Olink dataset (*n* = 33,995, Europeans only), resulting in a combined total of 59,176 participants. To account for kidney function in the full meta-analysis, including UK Biobank data where individual-level eGFR and UACR measurements were unavailable, we applied a summary-statistics-based conditional analysis as described by Wu et al. [51]. This method uses GWAS summary statistics and trait covariance estimates to approximate the joint model and thus efficiently adjust for kidney function without requiring individual-level data. To validate the adjustment approach, results based on summary statistics were compared with those derived from individual-level data where available. All meta-analyses and post-GWAS analyses were conducted based on the GRCh37/hg19 reference genome.

### Concordance between ELISA- and Olink-based GWAS results

To assess overall cross-platform consistency, we compared GWAS results for all SNPs shared between both datasets. Variants with MAF > 0.01 reaching *p* < 1 × 10^–06^ in at least one GWAS were included. Statistical similarity of effect sizes was evaluated using equivalence testing, applying a predefined equivalence margin of 0.333 times the phenotypic variance. As a more stringent sensitivity analysis, a smaller margin (Δ = 0.1) was additionally applied. Directional consistency and overlap of 95% confidence intervals (95% CI) were also evaluated. A scatter plot of β-estimates, including 95% CIs, was generated to visualize agreement, with both a line of identity (slope = 1) and a linear regression line to detect systematic bias.

While equivalence testing was applied to evaluate global cross-platform concordance, differences in effect estimates for specific variants, including conditionally independent loci, were assessed using z-tests to compute p_diff_ values using the following equation: $({\beta}_{UKB}-{\beta}_{ELISA})/\sqrt{S{E}_{UKB}^{2}+S{E}_{ELISA}^{2}}$. Multiple testing was addressed using Bonferroni correction based on the number of variants tested.

### Post-GWAS analyses

Visual quality control included QQ-plots and Manhattan plots, generated using custom R script. Unless otherwise stated, only variants with MAF > 0.001 were included in post-GWAS analyses. Locus regions were defined as ± 250 kb around the conditionally independent lead SNP of each region, where a genome-wide significant association was found and regional plots of the loci were generated using the LocalZoom platform (available at: https://statgen.github.io/localzoom/) [52]. Functional mapping and gene annotation of associated loci were performed using the UCSC Genome Browser [53] as well as using the FUMA platform (v1.5.2), available at https://fuma.ctglab.nl. [54]. The SNP2GENE function was used to perform gene mapping based on positional and eQTL annotations, and gene-based association analysis via MAGMA (v1.08). Subsequent biological interpretation was carried out using the GENE2FUNC module, which includes tissue-specific expression analysis (GTEx v8), gene-set enrichment (MsigDB), and overlap with genes reported in the GWAS Catalog. Additional lookups of conditionally independent SNPs were done using the Common Metabolic Diseases Knowledge Portal (SCR_020937, available at: https://hugeamp.org/ accessed March 13, 2025), which aggregates genetic association data across metabolic traits and diseases. Moreover, the FinnGen result browser was also used for this purpose (https://results.finngen.fi/ accessed June 10, 2025), which includes summary statistics for 2,469 traits based on 500,348 individuals. For sex-stratified analyses, we assessed sex-specific differences in genetic effects using a Z-test to compare effect sizes [55] between men and women in ELISA-based apoA-IV GWAS. Information on further post-GWAS analyses, including genetic correlation (Linkage Disequilibrium Score Regression [56, 57]) and colocalization analyses [58] as well as included summary statistics [51, 59–65] is provided in the supplemental materials.

## Supplementary Information

Additional file 1: Supplemental information on study populations and additional post-GWAS analyses as well as Figs. S1-S9.

Additional file 2: Tables S1-S8.

## Data Availability

GWAS summary statistics from the ELISA-only meta-analysis (basic model), the ELISA-only meta-analysis adjusted for eGFR, and the combined ELISA + UK Biobank meta-analysis are publicly available at: https://genepi.i-med.ac.at/data/apoa-iv-meta-gwas/ and Zenodo (https://doi.org/10.5281/zenodo.21023849) [66]. Relevant code used for the primary analyses is also available through this repository. Access to individual-level data from the contributing studies is subject to cohort-specific ethical approvals, consent restrictions, and data-sharing agreements. Researchers interested in accessing these data should contact the corresponding author, who can facilitate communication with the respective study investigators and provide guidance regarding the applicable access procedures and requirements. All additional results and tables supporting the conclusions of this article are included within the article and its supplemental materials.

## References

[1] Utermann G, Beisiegel U. Apolipoprotein A-IV: a protein occurring in human mesenteric lymph chylomicrons and free in plasma. Isolation and quantification Eur J Biochem. 1979;99(2):333–43. 10.1111/j.1432-1033.1979.tb13261.x 115688

[2] Kraft HG, Menzel HJ, Hoppichler F, Vogel W, Utermann G. Changes of genetic apolipoprotein phenotypes caused by liver transplantation. Implications for apolipoprotein synthesis. J Clin Invest. 1989;83(1):137–42. 10.1172/JCI113849 PMC303653 2521341

[3] Wang F, Kohan AB, Lo CM, Liu M, Howles P, Tso P. Apolipoprotein A-IV: a protein intimately involved in metabolism. J Lipid Res. 2015;56(8):1403–18. 10.1194/jlr.R052753 PMC4513983 25640749

[4] Kohan AB, Wang F, Lo CM, Liu M, Tso P. ApoA-IV: current and emerging roles in intestinal lipid metabolism, glucose homeostasis, and satiety. Am J Physiol Gastrointest Liver Physiol. 2015;308(6):G472-81. 10.1152/ajpgi.00098.2014 PMC4360046 25591862

[5] Wang F, Kohan AB, Kindel TL, Corbin KL, Nunemaker CS, Obici S, et al. Apolipoprotein A-IV improves glucose homeostasis by enhancing insulin secretion. Proc Natl Acad Sci U S A. 2012;109(24):9641–6. 10.1073/pnas.1201433109 PMC3386109 22619326

[6] Duverger N, Tremp G, Caillaud JM, Emmanuel F, Castro G, Fruchart JC, et al. Protection against atherogenesis in mice mediated by human apolipoprotein A-IV. Science. 1996;273(5277):966–8. 10.1126/science.273.5277.966 8688083

[7] Qu J, Ko CW, Tso P, Bhargava A. Apolipoprotein A-IV: a multifunctional protein involved in protection against atherosclerosis and diabetes. Cells. 2019. 10.3390/cells8040319. PMC6523623 30959835

[8] Kohan AB, Wang F, Li X, Bradshaw S, Yang Q, Caldwell JL, et al. Apolipoprotein A-IV regulates chylomicron metabolism-mechanism and function. Am J Physiol Gastrointest Liver Physiol. 2012;302(6):G628-36. 10.1152/ajpgi.00225.2011 PMC3311309 22207575

[9] Steinmetz A, Barbaras R, Ghalim N, Clavey V, Fruchart JC, Ailhaud G. Human apolipoprotein A-IV binds to apolipoprotein A-I/A-II receptor sites and promotes cholesterol efflux from adipose cells. J Biol Chem. 1990;265(14):7859–63. 2159462

[10] Stein O, Stein Y, Lefevre M, Roheim PS. The role of apolipoprotein A-IV in reverse cholesterol transport studied with cultured cells and liposomes derived from an ether analog of phosphatidylcholine. Biochim Biophys Acta. 1986;878(1):7–13. 10.1016/0005-2760(86)90337-1 3089295

[11] Qin X, Swertfeger DK, Zheng S, Hui DY, Tso P. Apolipoprotein AIV: a potent endogenous inhibitor of lipid oxidation. Am J Physiol. 1998;274(5):H1836–40. 10.1152/ajpheart.1998.274.5.H1836 9612397

[12] Xu XR, Wang Y, Adili R, Ju L, Spring CM, Jin JW, et al. Apolipoprotein A-IV binds αIIbβ3 integrin and inhibits thrombosis. Nat Commun. 2018;9(1):3608. 10.1038/s41467-018-05806-0 PMC6127106 30190457

[13] Kronenberg F, Stühlinger M, Trenkwalder E, Geethanjali FS, Pachinger O, von Eckardstein A, et al. Low apolipoprotein A-IV plasma concentrations in men with coronary artery disease. J Am Coll Cardiol. 2000;36(3):751–7. 10.1016/s0735-1097(00)00775-0 10987595

[14] Ezeh B, Haiman M, Alber HF, Kunz B, Paulweber B, Lingenhel A, et al. Plasma distribution of apoA-IV in patients with coronary artery disease and healthy controls. J Lipid Res. 2003;44(8):1523–9. 10.1194/jlr.M300060-JLR200 12777472

[15] Kollerits B, Krane V, Drechsler C, Lamina C, März W, Ritz E, et al. Apolipoprotein A-IV concentrations and clinical outcomes in haemodialysis patients with type 2 diabetes mellitus–a post hoc analysis of the 4D Study. J Intern Med. 2012;272(6):592–600. 10.1111/j.1365-2796.2012.02585.x 22891946

[16] Omori M, Watanabe M, Matsumoto K, Honda H, Hattori H, Akizawa T. Impact of serum apolipoprotein A-IV as a marker of cardiovascular disease in maintenance hemodialysis patients. Ther Apher Dial. 2010;14(3):341–8. 10.1111/j.1744-9987.2010.00809.x 20609189

[17] Stangl S, Kollerits B, Lamina C, Meisinger C, Huth C, Stöckl A, et al. Association between apolipoprotein A-IV concentrations and chronic kidney disease in two large population-based cohorts: results from the KORA studies. J Intern Med. 2015;278(4):410–23. 10.1111/joim.12380 26037138

[18] Kronenberg F, Kuen E, Ritz E, König P, Kraatz G, Lhotta K, et al. Apolipoprotein A-IV serum concentrations are elevated in patients with mild and moderate renal failure. J Am Soc Nephrol. 2002;13(2):461–9. 10.1681/ASN.V132461 11805176

[19] Kronenberg F. Apolipoprotein L1 and apolipoprotein A-IV and their association with kidney function. Curr Opin Lipidol. 2017;28(1):39–45. 10.1097/MOL.0000000000000371 27870653

[20] Olivier M, Wang X, Cole R, Gau B, Kim J, Rubin EM, et al. Haplotype analysis of the apolipoprotein gene cluster on human chromosome 11. Genomics. 2004;83(5):912–23. 10.1016/j.ygeno.2003.11.016 PMC2771640 15081120

[21] Lamina C, Friedel S, Coassin S, Rueedi R, Yousri NA, Seppälä I, et al. A genome-wide association meta-analysis on apolipoprotein A-IV concentrations. Hum Mol Genet. 2016;25(16):3635–46. 10.1093/hmg/ddw211 PMC5179953 27412012

[22] Sun BB, Chiou J, Traylor M, Benner C, Hsu YH, Richardson TG, et al. Plasma proteomic associations with genetics and health in the UK Biobank. Nature. 2023;622(7982):329–38. 10.1038/s41586-023-06592-6 PMC10567551 37794186

[23] Geronimo FRB, Barter PJ, Rye KA, Heather AK, Shearston KD, Rodgers KJ. Plaque stabilizing effects of apolipoprotein A-IV. Atherosclerosis. 2016;251:39–46. 10.1016/j.atherosclerosis.2016.04.019 27240254

[24] Schwaiger JP, Kollerits B, Steinbrenner I, Weissensteiner H, Schönherr S, Forer L, et al. Apolipoprotein A-IV concentrations and clinical outcomes in a large chronic kidney disease cohort: results from the GCKD study. J Intern Med. 2022;291(5):622–36. 10.1111/joim.13437 PMC9305919 34914850

[25] Kollerits B, Gruber S, Steinbrenner I, Schwaiger JP, Weissensteiner H, Schönherr S, et al. Apolipoprotein A-IV concentrations and cancer in a large cohort of chronic kidney disease patients: results from the GCKD study. BMC Cancer. 2024;24(1):320. 10.1186/s12885-024-12053-8 PMC10921727 38454416

[26] Boes E, Fliser D, Ritz E, König P, Lhotta K, Mann JF, et al. Apolipoprotein A-IV predicts progression of chronic kidney disease: the mild to moderate kidney disease study. J Am Soc Nephrol. 2006;17(2):528–36. 10.1681/ASN.2005070733 16382017

[27] Lingenhel A, Lhotta K, Neyer U, Heid IM, Rantner B, Kronenberg MF, et al. Role of the kidney in the metabolism of apolipoprotein A-IV: influence of the type of proteinuria. J Lipid Res. 2006;47(9):2071–9. 10.1194/jlr.M600178-JLR200 16788210

[28] Mack S, Coassin S, Vaucher J, Kronenberg F, Lamina C, Consortium A-I-G. Evaluating the causal relation of ApoA-IV with disease-related traits - a bidirectional two-sample Mendelian randomization study. Sci Rep. 2017;7(1):8734. 10.1038/s41598-017-07213-9 PMC5562707 28821713

[29] Kennedy AE, Ozbek U, Dorak MT. What has GWAS done for HLA and disease associations? Int J Immunogenet. 2017;44(5):195–211. 10.1111/iji.12332 28877428

[30] Trowsdale J, Knight JC. Major histocompatibility complex genomics and human disease. Annu Rev Genom Hum Genet. 2013;14:301–23. 10.1146/annurev-genom-091212-153455 PMC4426292 23875801

[31] Lhotta K. Uromodulin and chronic kidney disease. Kidney Blood Press Res. 2010;33(5):393–8. 10.1159/000320681 20948228

[32] Lamina C, Coassin S, Illig T, Kronenberg F. Look beyond one’s own nose: combination of information from publicly available sources reveals an association of GATA4 polymorphisms with plasma triglycerides. Atherosclerosis. 2011;219(2):698–703. 10.1016/j.atherosclerosis.2011.08.044 21945496

[33] Bideyan L, López Rodríguez M, Priest C, Kennelly JP, Gao Y, Ferrari A, et al. Hepatic GATA4 regulates cholesterol and triglyceride homeostasis in collaboration with LXRs. Genes Dev. 2022;36(21–24):1129–44. 10.1101/gad.350145.122 PMC9851399 36522129

[34] Asselbergs FW, Guo Y, van Iperen EP, Sivapalaratnam S, Tragante V, Lanktree MB, et al. Large-scale gene-centric meta-analysis across 32 studies identifies multiple lipid loci. Am J Hum Genet. 2012;91(5):823–38. 10.1016/j.ajhg.2012.08.032 PMC3487124 23063622

[35] Schönherr S, Schachtl-Riess JF, Di Maio S, Filosi M, Mark M, Lamina C, et al. Performing highly parallelized and reproducible GWAS analysis on biobank-scale data. NAR Genom Bioinform. 2024;6(1):lqae015. 10.1093/nargab/lqae015 PMC10849172 38327871

[36] Holle R, Happich M, Löwel H, Wichmann HE, Group MKS. KORA--a research platform for population based health research. Gesundheitswesen. 2005;67(Suppl 1):S19-25. 10.1055/s-2005-858235 16032513

[37] Brandl C, Zimmermann ME, Günther F, Barth T, Olden M, Schelter SC, et al. On the impact of different approaches to classify age-related macular degeneration: results from the German AugUR study. Sci Rep. 2018;8(1):8675. 10.1038/s41598-018-26629-5 PMC5989235 29875478

[38] Stark K, Olden M, Brandl C, Dietl A, Zimmermann ME, Schelter SC, et al. The German AugUR study: study protocol of a prospective study to investigate chronic diseases in the elderly. BMC Geriatr. 2015;15:130. 10.1186/s12877-015-0122-0 PMC4617905 26489512

[39] Titze S, Schmid M, Köttgen A, Busch M, Floege J, Wanner C, et al. Disease burden and risk profile in referred patients with moderate chronic kidney disease: composition of the German Chronic Kidney Disease (GCKD) cohort. Nephrol Dial Transplant. 2015;30(3):441–51. 10.1093/ndt/gfu294 25271006

[40] Fazzini F, Lamina C, Fendt L, Schultheiss UT, Kotsis F, Hicks AA, et al. Mitochondrial DNA copy number is associated with mortality and infections in a large cohort of patients with chronic kidney disease. Kidney Int. 2019;96(2):480–8. 10.1016/j.kint.2019.04.021 31248648

[41] Firmann M, Mayor V, Vidal PM, Bochud M, Pécoud A, Hayoz D, et al. The CoLaus study: a population-based study to investigate the epidemiology and genetic determinants of cardiovascular risk factors and metabolic syndrome. BMC Cardiovasc Disord. 2008;8:6. 10.1186/1471-2261-8-6 PMC2311269 18366642

[42] Raitakari OT, Juonala M, Rönnemaa T, Keltikangas-Järvinen L, Räsänen L, Pietikäinen M, et al. Cohort profile: the cardiovascular risk in Young Finns study. Int J Epidemiol. 2008;37(6):1220–6. 10.1093/ije/dym225 18263651

[43] van Herpt TTW, Lemmers RFH, van Hoek M, Langendonk JG, Erdtsieck RJ, Bravenboer B, et al. Introduction of the DiaGene study: clinical characteristics, pathophysiology and determinants of vascular complications of type 2 diabetes. Diabetol Metab Syndr. 2017;9:47. 10.1186/s13098-017-0245-x PMC5477157 28649285

[44] Ikram MA, Brusselle G, Ghanbari M, Goedegebure A, Ikram MK, Kavousi M, et al. Objectives, design and main findings until 2020 from the Rotterdam study. Eur J Epidemiol. 2020;35(5):483–517. 10.1007/s10654-020-00640-5 PMC7250962 32367290

[45] Ikram MA, Kieboom BCT, Brouwer WP, Brusselle G, Chaker L, Ghanbari M, et al. The Rotterdam study. Design update and major findings between 2020 and 2024. Eur J Epidemiol. 2020;39(2):183–206. 10.1007/s10654-023-01094-1 38324224

[46] Kronenberg F, Lobentanz EM, König P, Utermann G, Dieplinger H. Effect of sample storage on the measurement of lipoprotein[a], apolipoproteins B and A-IV, total and high density lipoprotein cholesterol and triglycerides. J Lipid Res. 1994;35(7):1318–28. 7964193

[47] Mbatchou J, Barnard L, Backman J, Marcketta A, Kosmicki JA, Ziyatdinov A, et al. Computationally efficient whole-genome regression for quantitative and binary traits. Nat Genet. 2021;53(7):1097–103. 10.1038/s41588-021-00870-7 34017140

[48] Willer CJ, Li Y, Abecasis GR. METAL: fast and efficient meta-analysis of genomewide association scans. Bioinformatics. 2010;26(17):2190–1. 10.1093/bioinformatics/btq340 PMC2922887 20616382

[49] Yang J, Lee SH, Goddard ME, Visscher PM. GCTA: a tool for genome-wide complex trait analysis. Am J Hum Genet. 2011;88(1):76–82. 10.1016/j.ajhg.2010.11.011 PMC3014363 21167468

[50] Bycroft C, Freeman C, Petkova D, Band G, Elliott LT, Sharp K, et al. The UK Biobank resource with deep phenotyping and genomic data. Nature. 2018;562(7726):203–9. 10.1038/s41586-018-0579-z PMC6786975 30305743

[51] Wu P, Wang B, Lubitz SA, Benjamin EJ, Meigs JB, Dupuis J. Approximate conditional phenotype analysis based on genome wide association summary statistics. Sci Rep. 2021;11(1):2518. 10.1038/s41598-021-82000-1 PMC7843738 33510268

[52] Boughton AP, Welch RP, Flickinger M, VandeHaar P, Taliun D, Abecasis GR, et al. LocusZoom.js: Interactive and embeddable visualization of genetic association study results. Bioinformatics. 2021;37(18):3017–18. 10.1093/bioinformatics/btab186 PMC8479674 33734315

[53] Lee BT, Barber GP, Benet-Pagès A, Casper J, Clawson H, Diekhans M, et al. The UCSC Genome Browser database: 2022 update. Nucleic Acids Res. 2022;50(D1):D1115–22. 10.1093/nar/gkab959 PMC8728131 34718705

[54] Watanabe K, Taskesen E, van Bochoven A, Posthuma D. Functional mapping and annotation of genetic associations with FUMA. Nat Commun. 2017;8(1):1826. 10.1038/s41467-017-01261-5 PMC5705698 29184056

[55] Winkler TW, Justice AE, Cupples LA, Kronenberg F, Kutalik Z, Heid IM. Approaches to detect genetic effects that differ between two strata in genome-wide meta-analyses: recommendations based on a systematic evaluation. PLoS ONE. 2017;12(7):e0181038. 10.1371/journal.pone.0181038 PMC5531538 28749953

[56] Bulik-Sullivan BK, Loh PR, Finucane HK, Ripke S, Yang J, Patterson N, et al. LD Score regression distinguishes confounding from polygenicity in genome-wide association studies. Nat Genet. 2015;47(3):291–5. 10.1038/ng.3211 PMC4495769 25642630

[57] Altshuler DM, Gibbs RA, Peltonen L, Dermitzakis E, Schaffner SF, Yu F, et al. Integrating common and rare genetic variation in diverse human populations. Nature. 2010;467(7311):52–8. 10.1038/nature09298 PMC3173859 20811451

[58] Giambartolomei C, Vukcevic D, Schadt EE, Franke L, Hingorani AD, Wallace C, et al. Bayesian test for colocalisation between pairs of genetic association studies using summary statistics. PLoS Genet. 2014;10(5):e1004383. 10.1371/journal.pgen.1004383 PMC4022491 24830394

[59] Graham SE, Clarke SL, Wu KH, Kanoni S, Zajac GJM, Ramdas S, et al. The power of genetic diversity in genome-wide association studies of lipids. Nature. 2021;600(7890):675–9. 10.1038/s41586-021-04064-3 PMC8730582 34887591

[60] Stanzick KJ, Li Y, Schlosser P, Gorski M, Wuttke M, Thomas LF, et al. Discovery and prioritization of variants and genes for kidney function in >1.2 million individuals. Nat Commun. 2021;12(1):4350. 10.1038/s41467-021-24491-0 PMC8285412 34272381

[61] Manousaki D, Mitchell R, Dudding T, Haworth S, Harroud A, Forgetta V, et al. Genome-wide association study for vitamin D levels reveals 69 independent loci. Am J Hum Genet. 2020;106(3):327–37. 10.1016/j.ajhg.2020.01.017 PMC7058824 32059762

[62] Loya H, Kalantzis G, Cooper F, Palamara PF. A scalable variational inference approach for increased mixed-model association power. Nat Genet. 2025;57(2):461–8. 10.1038/s41588-024-02044-7 PMC11821521 39789286

[63] Cerezo M, Sollis E, Ji Y, Lewis E, Abid A, Bircan KO, et al. The NHGRI-EBI GWAS Catalog: standards for reusability, sustainability and diversity. Nucleic Acids Res. 2025;53(D1):D998-1005. 10.1093/nar/gkae1070 PMC11701593 39530240

[64] Teumer A, Li Y, Ghasemi S, Prins BP, Wuttke M, Hermle T, et al. Genome-wide association meta-analyses and fine-mapping elucidate pathways influencing albuminuria. Nat Commun. 2019;10(1):4130. 10.1038/s41467-019-11576-0 PMC6739370 31511532

[65] Jiang L, Zheng Z, Fang H, Yang J. A generalized linear mixed model association tool for biobank-scale data. Nat Genet. 2021;53(11):1616–21. 10.1038/s41588-021-00954-4 34737426

[66] Koller A, Kronenberg F. Meta-analysis of GWAS on apolipoprotein A-IV. 2026. Zenodo. 10.5281/zenodo.21023849.

