# Supplementary material for: Genetic architecture and cross-platform validation of apolipoprotein A-IV concentrations
Source: Genome Biol. 2026 Jul 14;27:307. doi: 10.1186/s13059-026-04196-7 (PMC13647689; doi:10.1186/s13059-026-04196-7)
Supplement: Supplementary file 1 [file 13059_2026_4196_MOESM1_ESM.pdf]

Supplemental Material for

Genetic architecture and cross-platform validation of apolipoprotein A-IV concentrations

Adriana Koller[1], Florian Schnitzer[1], Barbara Kollerits[1], Claudia Lamina[1], Samuel Moix[2], Annique Claringbould[3], Binisha H Mishra[4], Haojie Lu[3,5], Johanna F Schachtl-Riess[1], Lukas Forer[1], Christian Gieger[6,7,8, †], Azin Kheirkhah[1], Terho Lehtimäki[4], Pedro Marques-Vidal[9], Sebastian Schönherr[1], Klaus J Stark[10], Reinhard Würzner[11], Kai-Uwe Eckardt[12,13], Iris M Heid[10], Maryam Kavousi[5], Anna Köttgen[14], Olli Raitakari[15,16,17,18], Eric J G Sijbrands[3], Annette Peters[6], Peter Vollenweider[9], Florian Kronenberg[1]

[1] Institute of Genetic Epidemiology, Medical University of Innsbruck, Innsbruck, Austria
[2] Department of Computational Biology, University of Lausanne, Lausanne, Switzerland; Swiss Institute of Bioinformatics, Lausanne, Switzerland; University Center for Primary Care and Public Health, Lausanne, Switzerland
[3] Department of Internal Medicine, Erasmus University Medical Center, Rotterdam, The Netherlands
[4] Department of Clinical Chemistry, Fimlab Laboratories and Finnish Cardiovascular Research Center-Tampere, Faculty of Medicine and Health Technology, Tampere University, Tampere, Finland
[5] Department of Epidemiology, Erasmus University Medical Center Rotterdam, Rotterdam, The Netherlands
[6] Institute of Epidemiology, Helmholtz Zentrum München, German Research Center for Environmental Health (GmbH), Neuherberg, Germany
[7] Research Unit Molecular Epidemiology, Helmholtz Zentrum München, German Research Center for Environmental Health (GmbH), Neuherberg, Germany.
[8] German Center for Diabetes Research (DZD), Neuherberg, Germany.
[9] Department of Medicine, Internal Medicine, Lausanne University Hospital (CHUV), and University of Lausanne, Lausanne, Switzerland
[10] Department of Genetic Epidemiology, University of Regensburg, Regensburg, Germany
[11] Institute of Hygiene and Medical Microbiology, Medical University of Innsbruck, Innsbruck, Austria
[12] Department of Nephrology and Hypertension, University Hospital Erlangen, Friedrich-Alexander-Universität Erlangen-Nürnberg, Erlangen, Germany
[13] Department of Nephrology and Medical Intensive Care, Charité – Universitätsmedizin Berlin, Berlin, Germany
[14] Institute of Epidemiology and Prevention, Faculty of Medicine and Medical Center - University of Freiburg, Freiburg, Germany
[15] Centre for Population Health Research, University of Turku and Turku University Hospital, Turku, Finland
[16] Research Centre of Applied and Preventive Cardiovascular Medicine, University of Turku, Turku, Finland
[17] Department of Clinical Physiology and Nuclear Medicine, Turku University Hospital, Turku, Finland
[18] InFLAMES Research Flagship, University of Turku, Turku, Finland

[†] Deceased during manuscript revision. We gratefully acknowledge the contributions of Prof. Christian Gieger, who passed away during manuscript revision and whose longstanding collaboration significantly contributed to this work.

Corresponding author:

Florian Kronenberg, MD
Institute of Genetic Epidemiology
Medical University of Innsbruck
Schöpfstrasse 3, A-6020 Innsbruck, Austria.
Phone: (+43) 512-9003-70560
Email: Florian.Kronenberg@i-med.ac.at

Supplementary Tables

Table S1: Genotyping information of all individuals included in the meta-analysis, separated by study.

Table S2: Baseline characteristics of individual studies included in the apoA-IV GWAS.

Table S3: Four conditionally independent top hits identified in the ELISA-based meta-analysis (n = 25,181) for each study separately.

Table S4: All conditionally independent top hits identified in the combined meta-analysis (n = 59,176) for each study separately.

Table S5: Adjusted beta estimates, and p-values for apoA-IV GWAS results after kidney function adjustment (eGFR or UACR) or diabetes across all studies (n = 59,176).

Table S6: Leave-one-cohort-out meta-analyses (fixed effects) for lead variants, with disease-enriched cohorts (GCKD and DiaGene) highlighted in blue.

Table S7: Significant genes identified by MAGMA gene-based testing, with input SNPs mapped to 20,043 protein-coding genes (genome-wide significance threshold: $P = 0.05 / 20{,}043 = 2.495 \times 10^{-06}$).

Table S8: List of 34 Lead SNPs from FUMA LD Clumping Based on Genome-Wide Significant Associations ($r^2 < 0.1$)

Study Populations

KORA studies

The Cooperative Health Research in the Region of Augsburg (KORA) F3 and F4 studies include 3,075 and 2,926 samples from the general population, respectively. The KORA F3 study, conducted in 2004/05, is a population-based sample from the general population in Augsburg, Southern Germany. All participants in the KORA F3 study have genome-wide data available, obtained using Illumina Omni 2.5/Illumina Omni Express. The KORA F4 survey, conducted in 2006-2008, is an independent sample from the same population (36). Genome-wide data are also available for all participants in the KORA F4 study, obtained using Affymetrix Axiom. GWAS was performed separately for KORA F3 and KORA F4.

AugUR study

The AugUR study (Altersbezogene Untersuchungen zur Gesundheit der University of Regensburg) is a population-based cohort study of the elderly population of Regensburg (Germany) to investigate age-related traits at the genetic and non-genetic levels. Details of the study design and data collection have been described elsewhere (37, 38). Briefly, 2,449 participants with at least 70 years of age at the time of sample and data collection were included. Information on sociodemographic data, lifestyle, metabolic parameters, medication intake, and morbidities was collected. The recruitment phase was split in two parts, henceforth referred to as “AugUR1” (n = 1,133) and “AugUR2” (n = 1,316) study. GWAS was performed separately for AugUR1 and AugUR2.

GCKD study

The German Chronic Kidney Disease (GCKD) study is a previously described prospective cohort study of 5,217 adult patients with chronic kidney disease (reduced kidney function and/or proteinuria) under regular care by nephrologists (39, 40). Trained personnel obtained information on socio-demographic factors, medical and family history, medications and health-related quality of life through standardized questionnaires. Data were collected and managed using the cloud-based web platform Askimed (https://www.askimed.com).

CoLaus

The CoLaus study is a cross-sectional study including 6,733 individuals at baseline aged 35-75 years from a single-center in Lausanne, Switzerland. Details on the study design have been described elsewhere. (41) The study aimed to investigate the epidemiology and genetic determinants of cardiovascular risk factors. Genotype data and phenotypes were available from 3,543 individuals.

Young Finns Study

The Cardiovascular Risk in Young Finns (YFS) started to recruit children (age: 3-18 years at baseline visit) and initiated several follow-ups since then (42). Several follow-ups were conducted over decades to investigate childhood risk factors for cardiovascular outcomes over time. From 2,103 individuals, both phenotype data as well as genotype data were available.

DiaGene Study

The multi-center, prospective DiaGene study includes 1886 patients with type 2 diabetes and 854 diabetes-free individuals with a mean age of 65 years recruited in Eindhoven, the Netherlands. Details on the study design have been previously published. (43)

Rotterdam Study

The Rotterdam Study is a population-based study of individuals living in Rotterdam, the Netherlands aged 40 years and over. Details on the study design have been described previously. (44, 45) Briefly, baseline investigations have started 1990 and major aims of this study are to unravel etiology, preclinical course, natural history and potential targets for intervention for diseases in mid- and late-life. GWAS was performed separately for Rotterdam Study 1 and 2.

UK Biobank

To validate findings in an independent study using a different approach for protein quantification, data from the UK Biobank (UKB) is incorporated, which utilized the antibody-based Olink Explore 3072 PEA technology. This analysis identified 2,923 distinct proteins, including apoA-IV. ApoA-IV levels are available for 44,852 individuals (22).

Additional post-GWAS analyses

Genetic Correlation Analysis

To investigate the genetic correlation between apoA-IV and lipid- and kidney-related traits as well as additional relevant blood parameters, we applied Linkage Disequilibrium Score Regression (LDSC) (56), which estimates the genome-wide genetic correlation and thus provides an overall measure of shared genetic etiology between apoA-IV and these traits of interest. The traits that were used in this analysis were selected based on the findings from the post-GWAS lookup for loci in several databases (see paragraph “post-GWAS analyses” in the main manuscript) as well as based on biological reasoning.

LDSC was performed with the ldscr package version 0.1.0 in R (https://github.com/mglev1n/ldscr/) using the built-in LD score reference files suitable for European ancestry individuals derived from the Pan-UK Biobank. Since the applied LD scores are only applicable for individuals of European ancestry, this analysis was conducted only with the European subset of the apoA-IV summary statistics. Additional information on the used summary statistics for the traits of interest can be found below.

As recommended by the LDSC developers, we restricted our analysis to common (MAF > 1%) single nucleotide variants and HapMap3 (57) SNPs. Moreover, variants with a sample size less than 0.67 × 90th percentile of the overall sample size were excluded. For summary statistics without per-SNP sample size, we used the total sample size. If the GWAS summary statistics did not already contain rsIDs, then this information was added using the "annotate" command of genomic-utils (https://github.com/genepi/genomic-utils). This annotation was performed using rsID reference data from pheweb (https://resources.pheweb.org/rsids-v154-hg19.tsv.gz) and with the "CHROM_POS_ALLELES" strategy (e.g. taking alleles into account).

The z-score of the effect associated with the alternative/effect allele (A1) was calculated by dividing the beta (effect size) by its standard error for each SNP in all summary statistics.

Colocalization Analysis

Regions around the lead SNP of each identified locus (±250kB) were investigated for colocalization of GWAS signals between apoA-IV and lipid- and kidney-related traits as well as additional relevant blood parameters using Approximate Bayes Factor (ABF) colocalization analysis (58).

ABF was performed with the coloc.abf() function of the R package coloc version 5.2.3 using the same GWAS summary statistics as in the LDSC analysis but without any filtering except for removal of SNPs with missing or duplicate rsIDs and of SNPs with a MAF of zero. Annotation of rsIDs was executed with genomic-utils as described above. In general, colocalization analysis was only performed if both traits showed at least one variant with a p-value < 10^{-6} in the examined region.

The standard deviation of the trait (sdY) was explicitly set to 1 for all inverse-normal transformed traits (all except vitamin D, $eGFR_{crea}$, and $eGFR_{cys}$). For the other traits sdY was estimated by coloc based on the minor allele frequency (MAF) and the sample size (N). We applied coloc to estimate the posterior probabilities (PP) for different hypotheses regarding the genetic association patterns between traits. In this study, we focused on hypothesis H4 (both traits are associated and share a single causal variant) and considered colocalization to be present if the posterior probability for H4 exceeded 80%.

Summary statistics used for the LDSC, colocalization analyses and for adjustment of summary statistics

The European-ancestry specific GWAS summary statistics for HDL-C, LDL-C and triglycerides were obtained from the Global Lipids Genetics Consortium (n~1,320,000, respectively). (59) Kidney function traits—eGFR based on serum creatinine and cystatin C—were based on summary statistics from Stanzick *et al.* (n = 1,201,909), accessed via the GWAS Catalog accession IDs GCST90103633-GCST90103635. (60) Summary statistics for serum 25-hydroxyvitamin D (n = 401,460) were provided by Manousaki *et al*. (GWAS Catalog accession IDs GCST010144). (61) Additional traits including

hemoglobin, blood cell counts, urate, BMI, serum albumin, HbA1c, sex hormone-binding globulin, and high-sensitivity C-reactive protein (hsCRP) were obtained from Loya et al. (GWAS Catalog accession IDs: accession numbers GCST90468059–GCST90471107) and derived from UK Biobank data (n = 405,088). (62) All summary statistics were downloaded from the NHGRI-EBI GWAS Catalog (63) on 31/03/2025.

Adjustment of summary statistics (Additional file 2: Table S5) was based on Wu *et al.* (51) (using reference data for log-transformed $eGFR_{crea}$ (GWAS catalog accession ID: GCST90103634) (60) and log-transformed UACR (GWAS catalog accession ID: GCST008794) (64)) as well as Jiang *et al. (*using reference data for diabetes (GWAS catalog accession ID: GCST90043636)) (65).

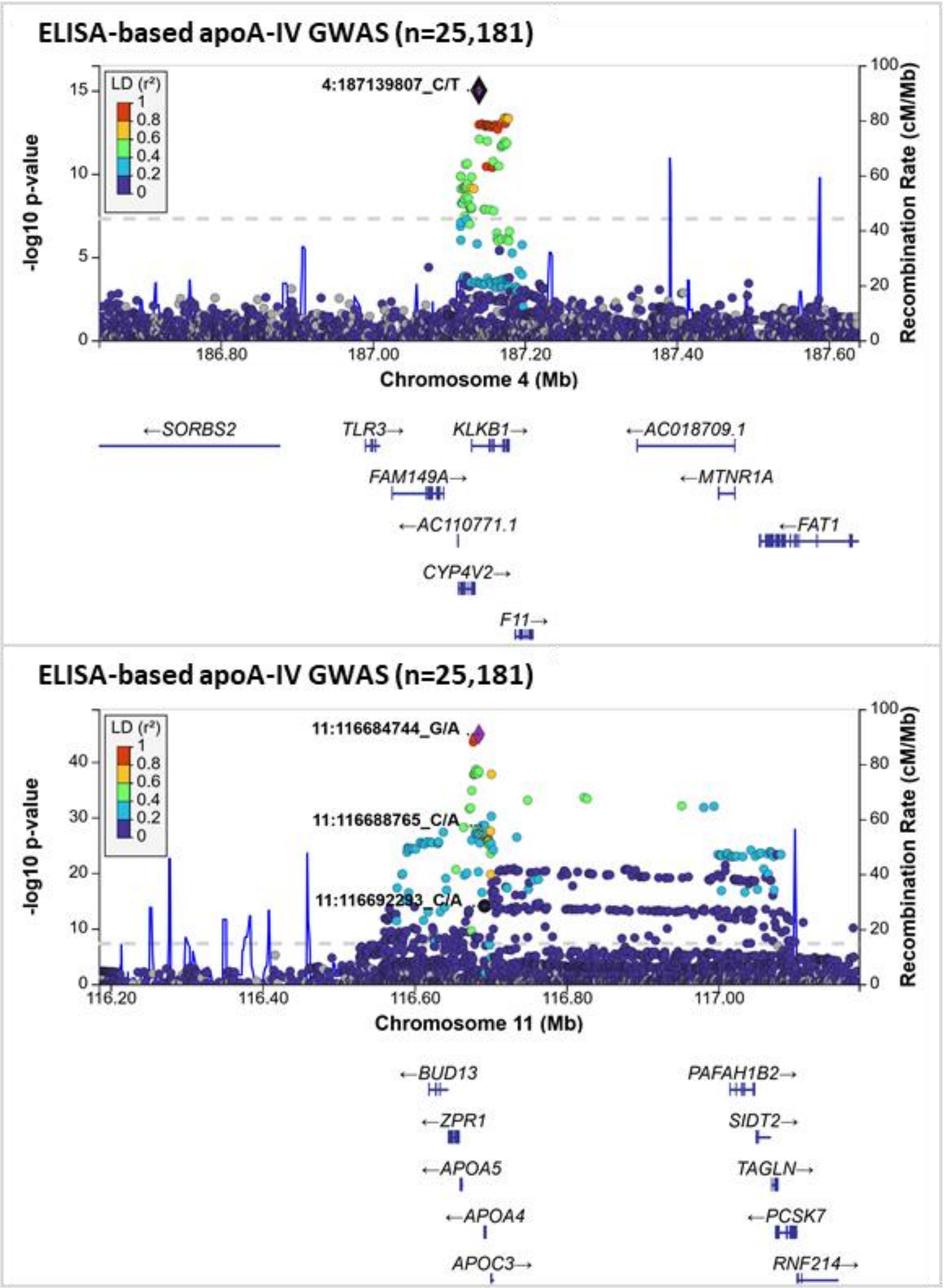

Fig S1: Regional LocalZoom plots for apoA-IV meta-GWAS including ELISA-based studies (n=25,181), with the conditionally independent index variant(s) labeled.

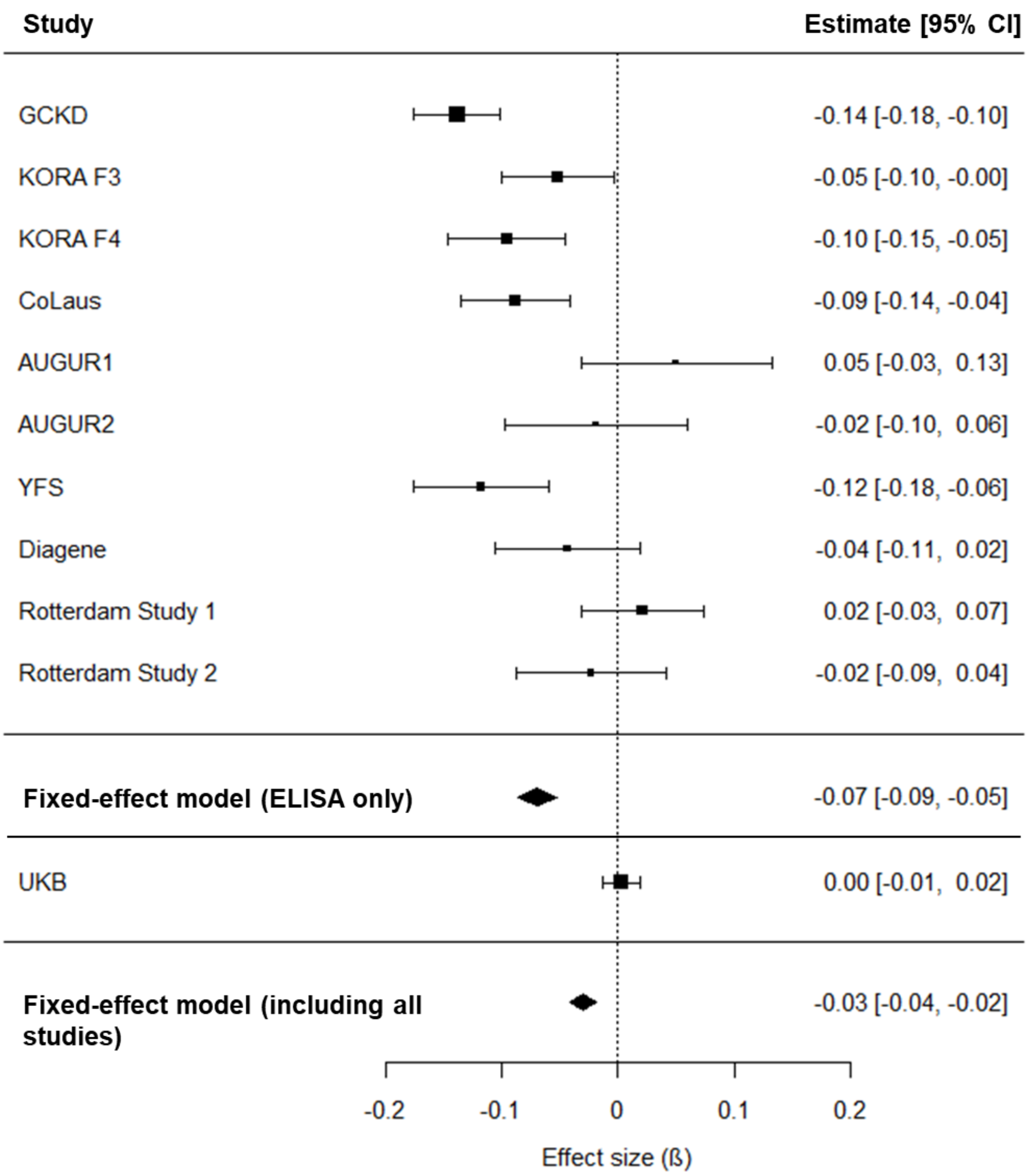

Fig S2: Forest plot of study-specific and pooled effect estimates (fixed effects model) for the lead SNP in the *KLKB1* locus (rs12331051). The upper panel shows the meta-analysis of the first 10 studies (ELISA-based cohorts), with the corresponding pooled estimate. The lower panel presents the additional study (UKB) and the overall pooled estimate incorporating all 11 studies. Study-level β estimates with 95% confidence intervals are shown for each contributing cohort. Horizontal lines indicate 95% confidence intervals. Diamonds represent the pooled effects estimates.

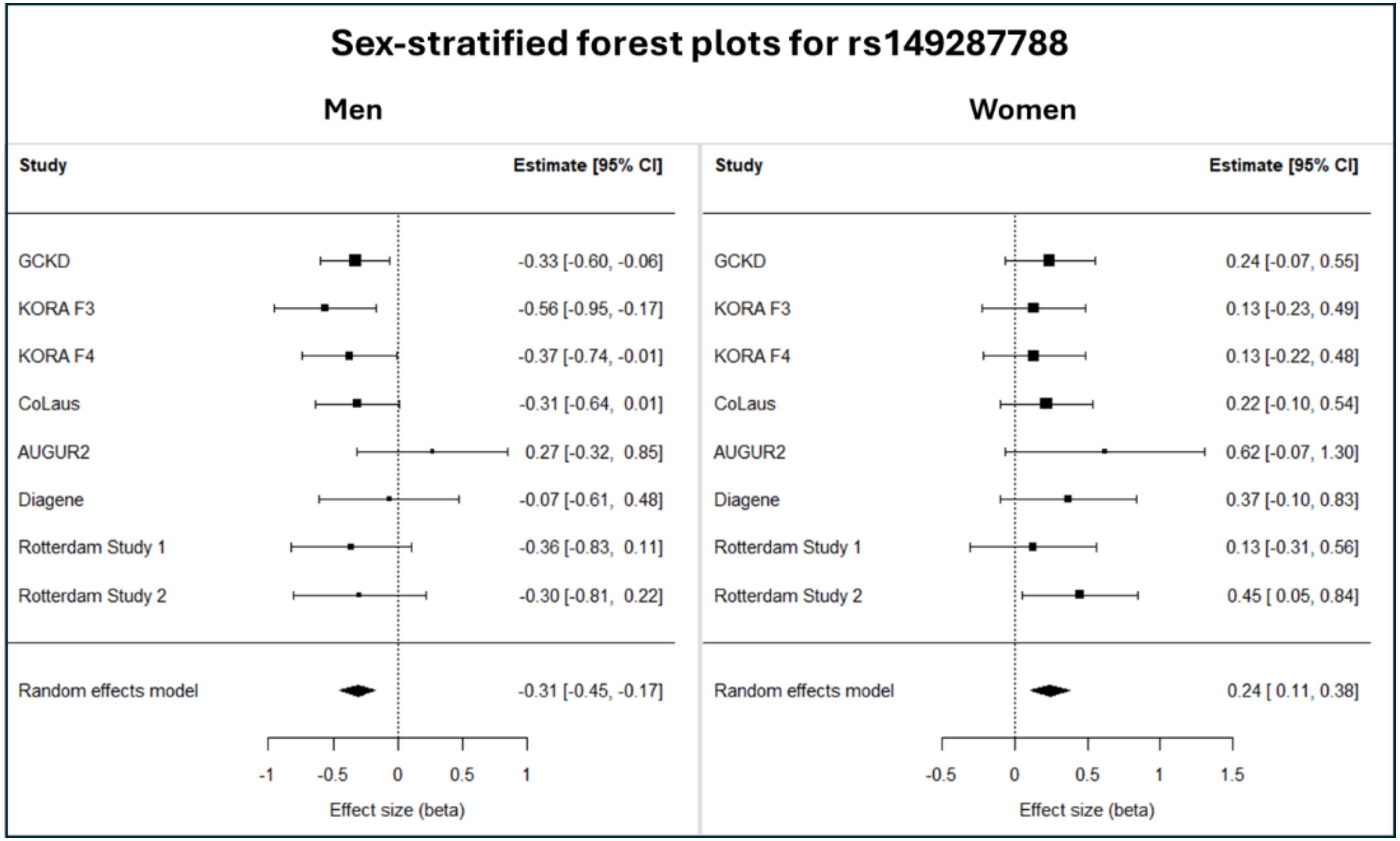

Fig S3. Sex-stratified forest plot for rs149287788 near *GSG1L*. Forest plot showing study-specific and meta-analytic effect estimates for rs149287788 (effect allele A) on apoA-IV levels in men and women. A Z-test comparing effect estimates between sexes identified a significant sex-differential association (P-value of difference = 2.44×10^{-09}), with a negative effect in men (β = -0.342) and a positive effect in women (β = +0.242). Minor allele frequencies were highly similar between men and women (0.0086 vs. 0.0087), and sample sizes were well balanced (n = 10,951 men; n = 10,910 women). Imputation quality was consistently high across cohorts (INFO > 0.8). Study-specific results demonstrate concordant effect directions across cohorts, indicating that the observed sex-differential association is not driven by a single study. Error bars represent 95% confidence intervals.

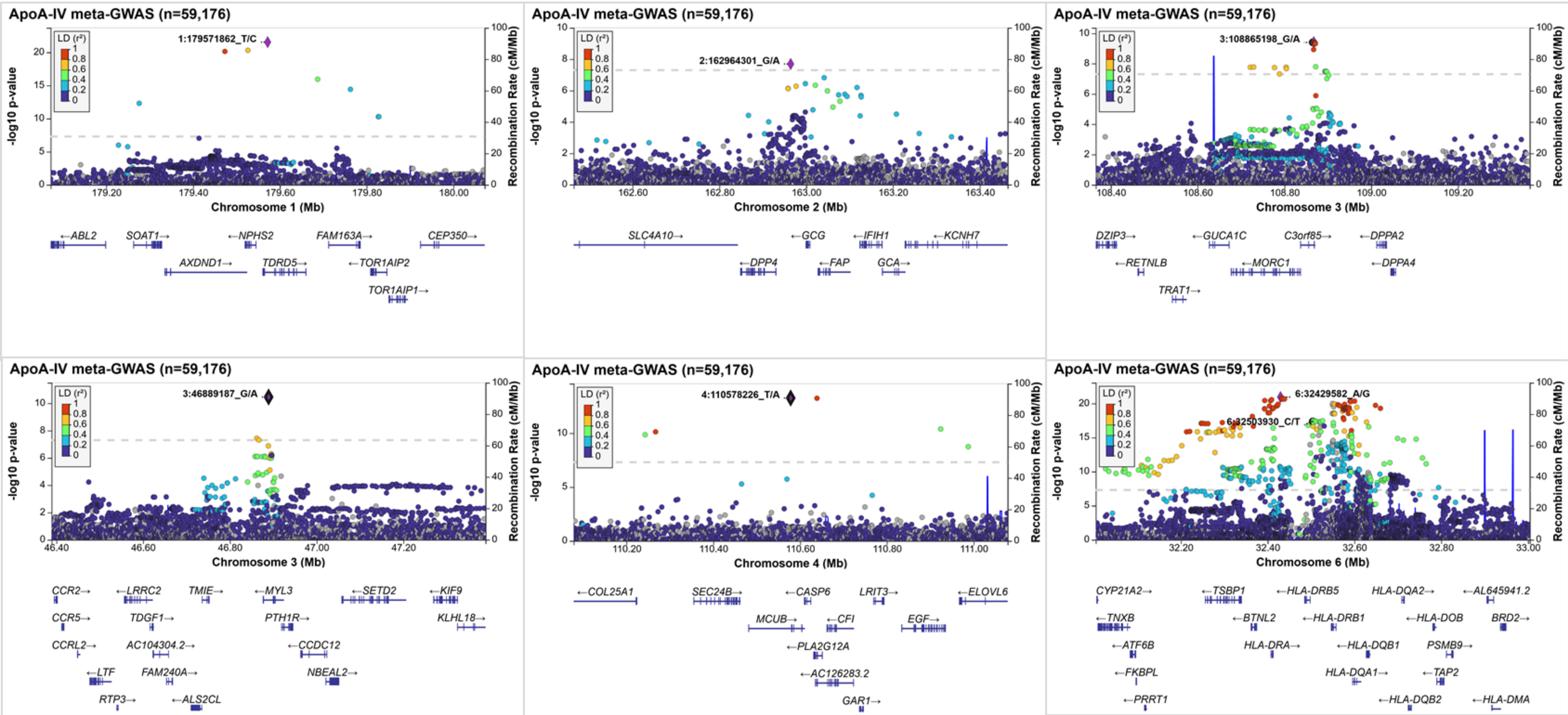

Fig S4: Regional LocalZoom plots for apoA-IV meta-GWAS including ELISA-based studies and UKB data, with the index variant(s) labeled.

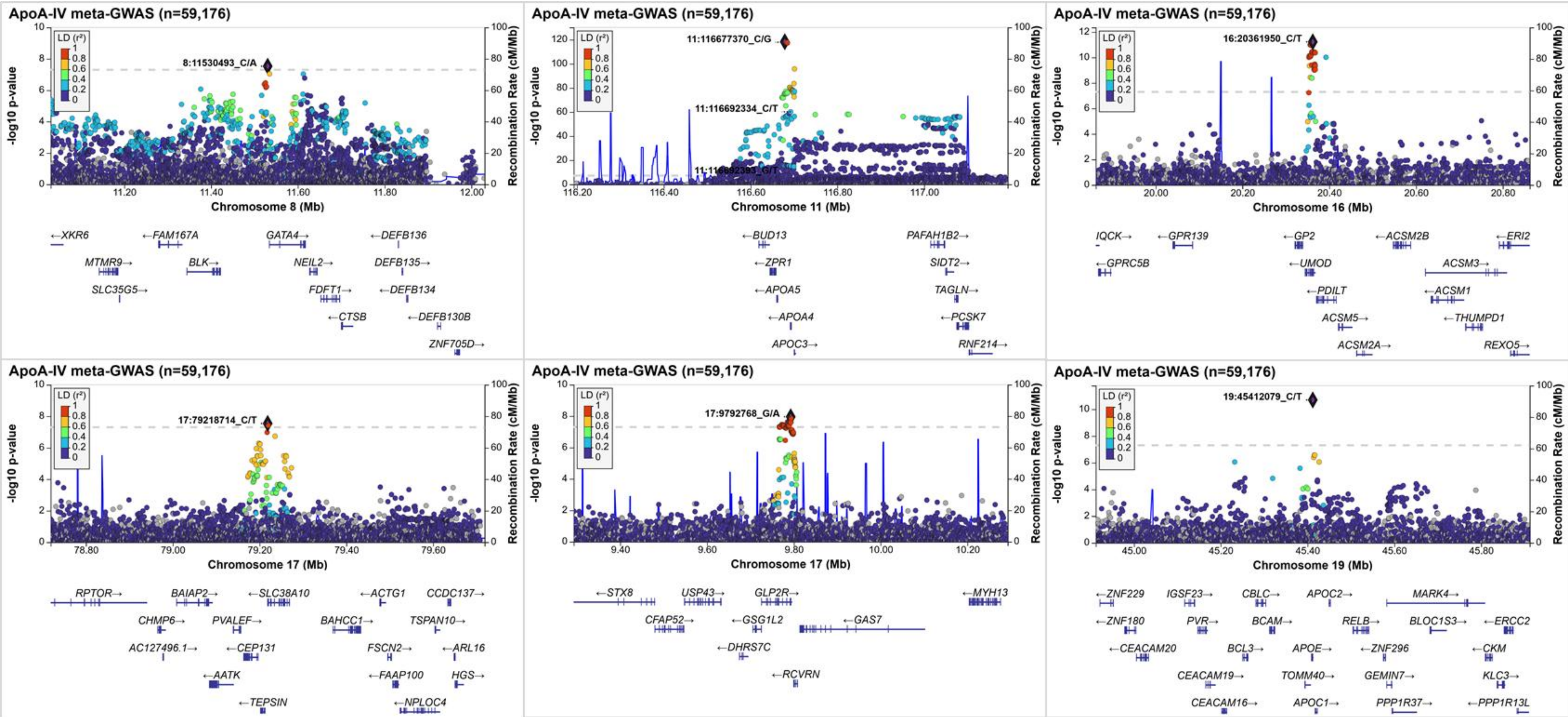

Fig S4 (continued)

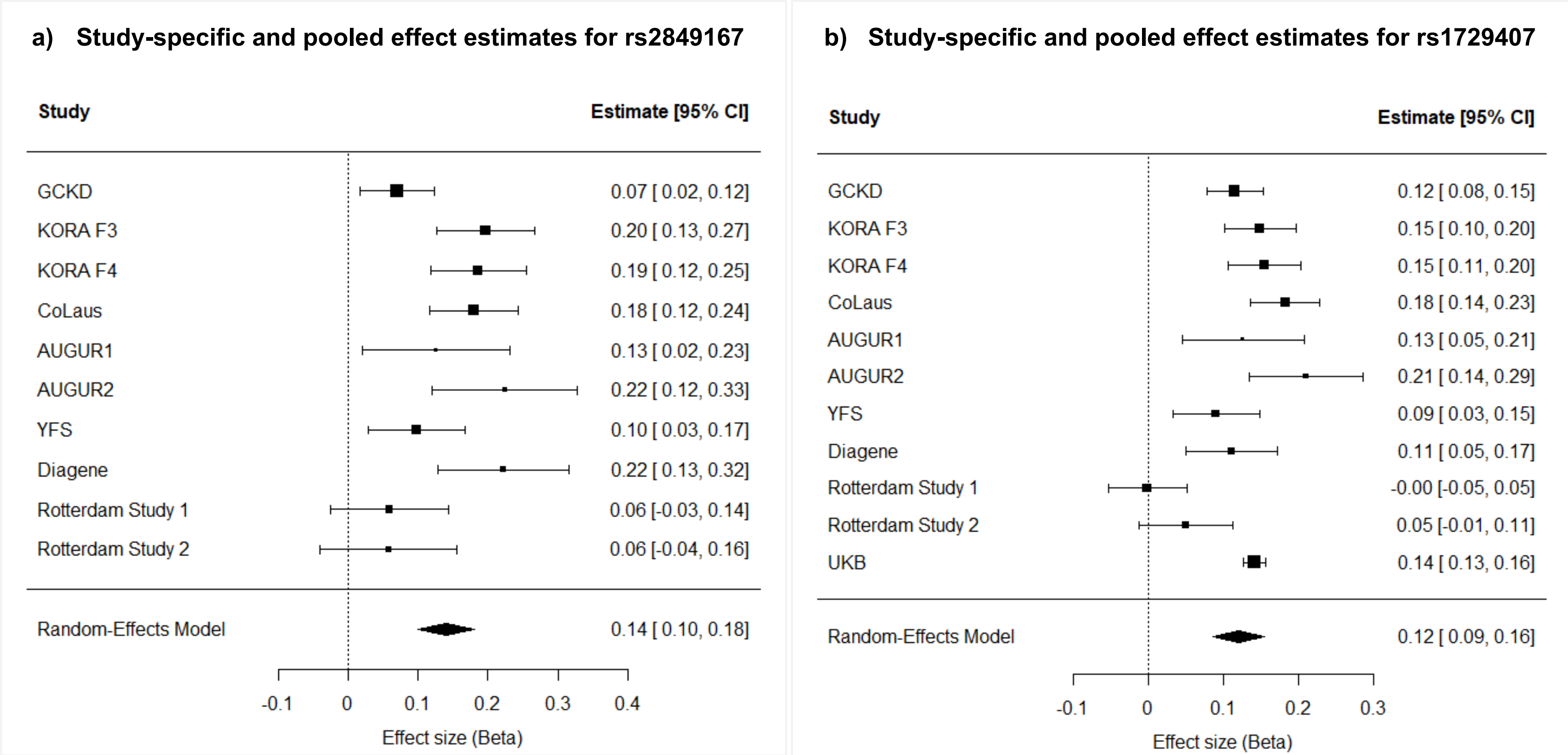

Fig S5: Forest plot of study-specific and pooled effect estimates (random effects model) for the lead SNPs (a) rs2849167 in the ELISA-based meta-analysis, and (b) rs1729407 in the entire meta-analysis including also UKB). Study-level β estimates with 95% confidence intervals are shown for each contributing cohort. Black squares represent the point estimates, with square size proportional to study weight. Horizontal lines indicate 95% confidence intervals. The diamond at the bottom represents the random-effects pooled estimate (β = 0.14, 95% CI: 0.10–0.18 and β = 0.12, 95% CI: 0.09–0.16, respectively). While heterogeneity across studies was considerable (with I^2 ~ 70-80%, $p < 0.0001$), the overall direction of effect was consistent across almost all cohorts, with variability mainly in effect magnitude.

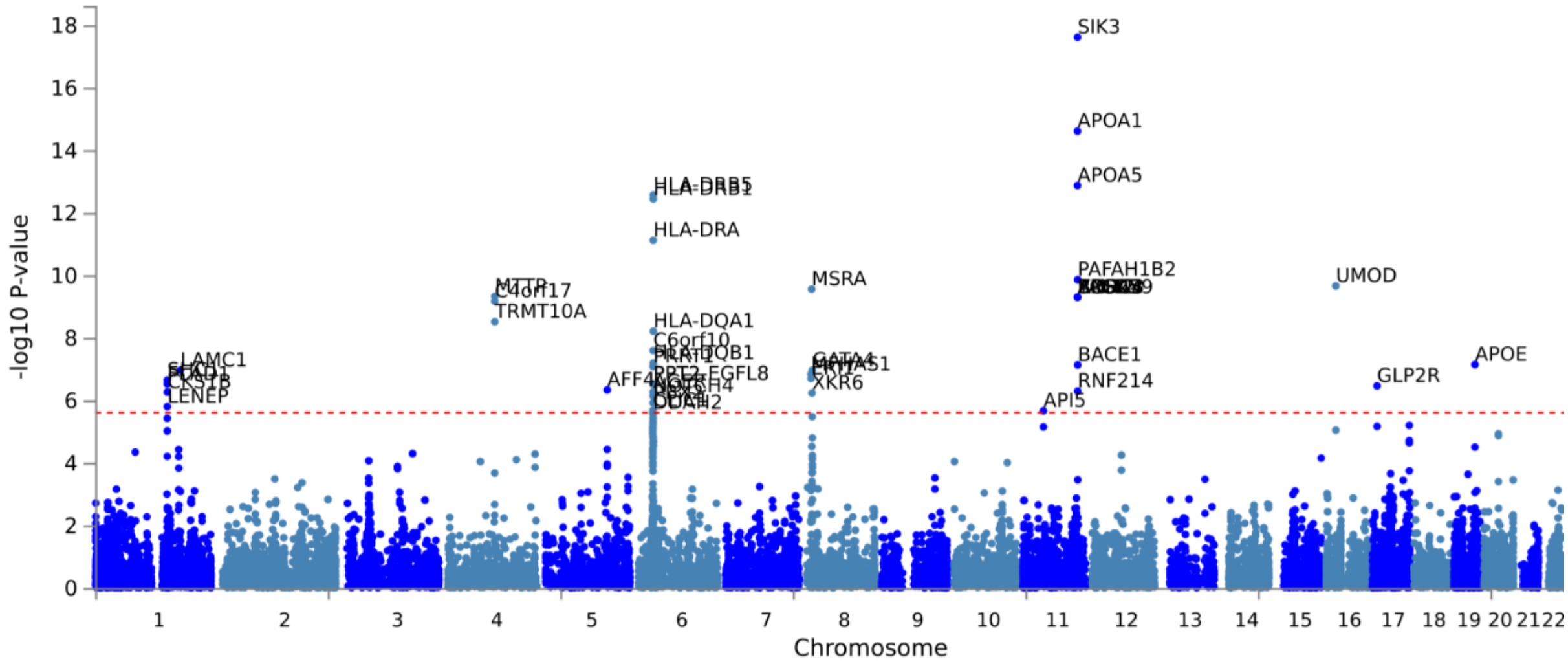

Fig S6: Manhattan plot of gene-based association results computed using MAGMA, mapping input SNPs to 20,043 protein-coding genes. The red dashed line indicates genome-wide significance (P < 2.495×10^{-06} (0.05/20043)). Analyses were conducted using FUMA SNP2GENE/GENE2FUNC tools, based on genome-wide association results for apoA-IV concentrations, including the combined meta-analysis (n = 59,176). While some gene names overlap in the plot, the full list of significant genes is provided in *Table S6*.

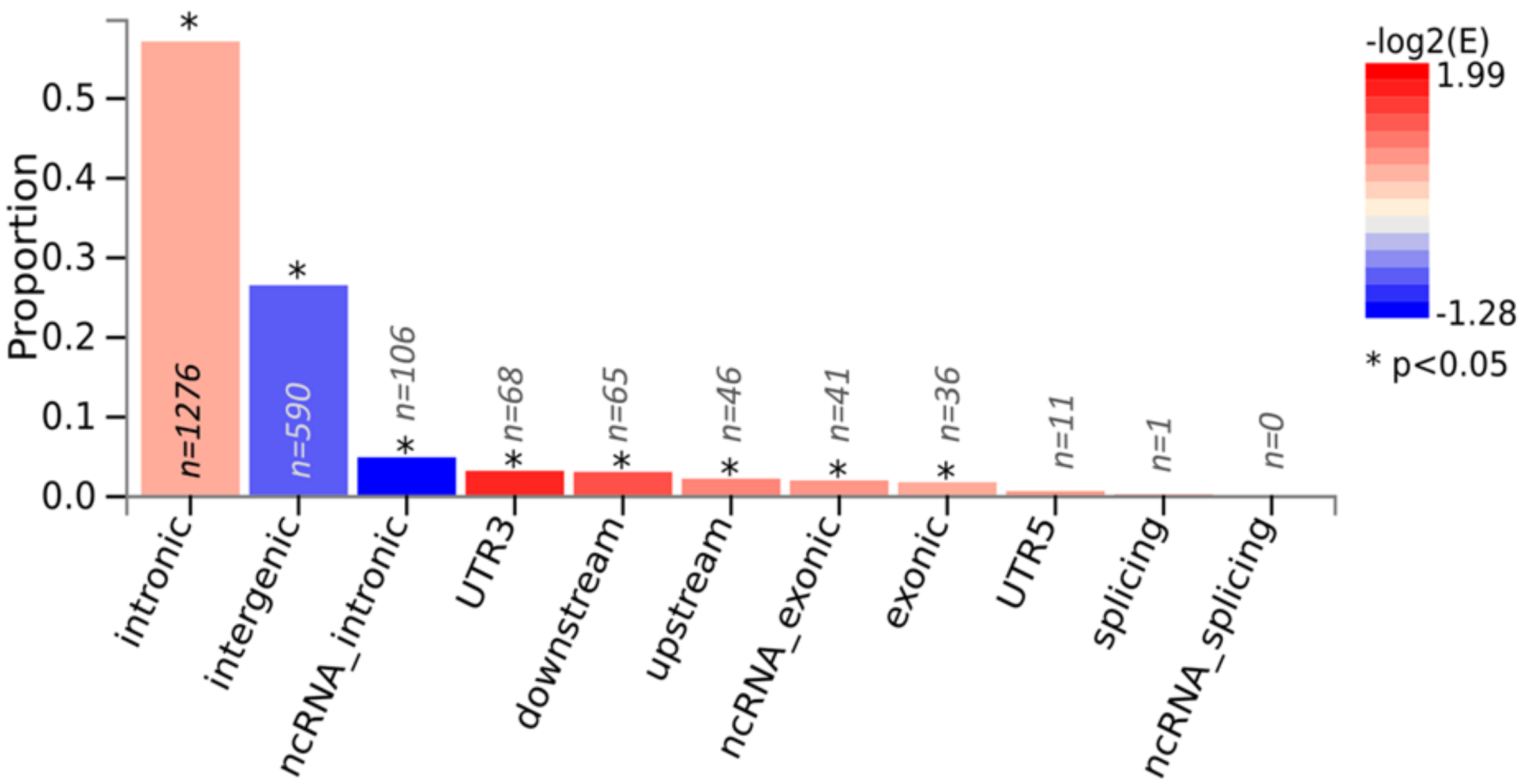

Fig S7: Functional annotation of SNPs, showing the distribution and enrichment (log_2 scale) of predicted functional consequences from ANNOVAR for SNPs in LD with lead SNPs (125 lead SNPs were identified through FUMA LD-based clumping). Analyses were conducted using FUMA SNP2GENE/GENE2FUNC tools, based on genome-wide association results for apoA-IV concentrations, including the combined meta-analysis (n = 59,176).

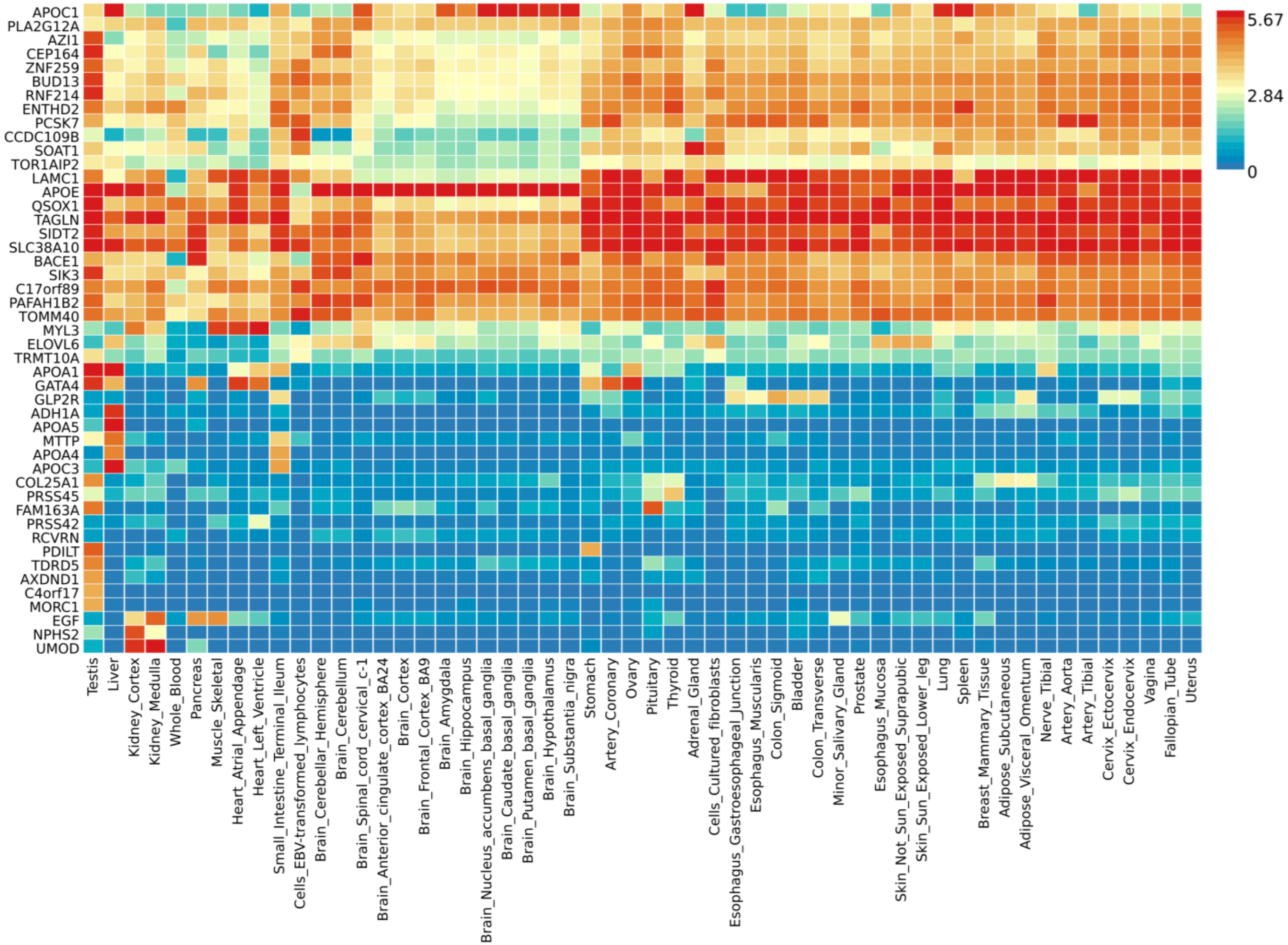

Fig S8: Gene expression heatmap based on GTEx v8, displaying average log_2-transformed expression levels of mapped genes. Analyses were conducted using FUMA SNP2GENE/GENE2FUNC tools, based on genome-wide association results for apoA-IV concentrations, including the combined meta-analysis (n = 59,176).

A) GWAS on apoA-IV vs. triglycerides in the *APOE* region

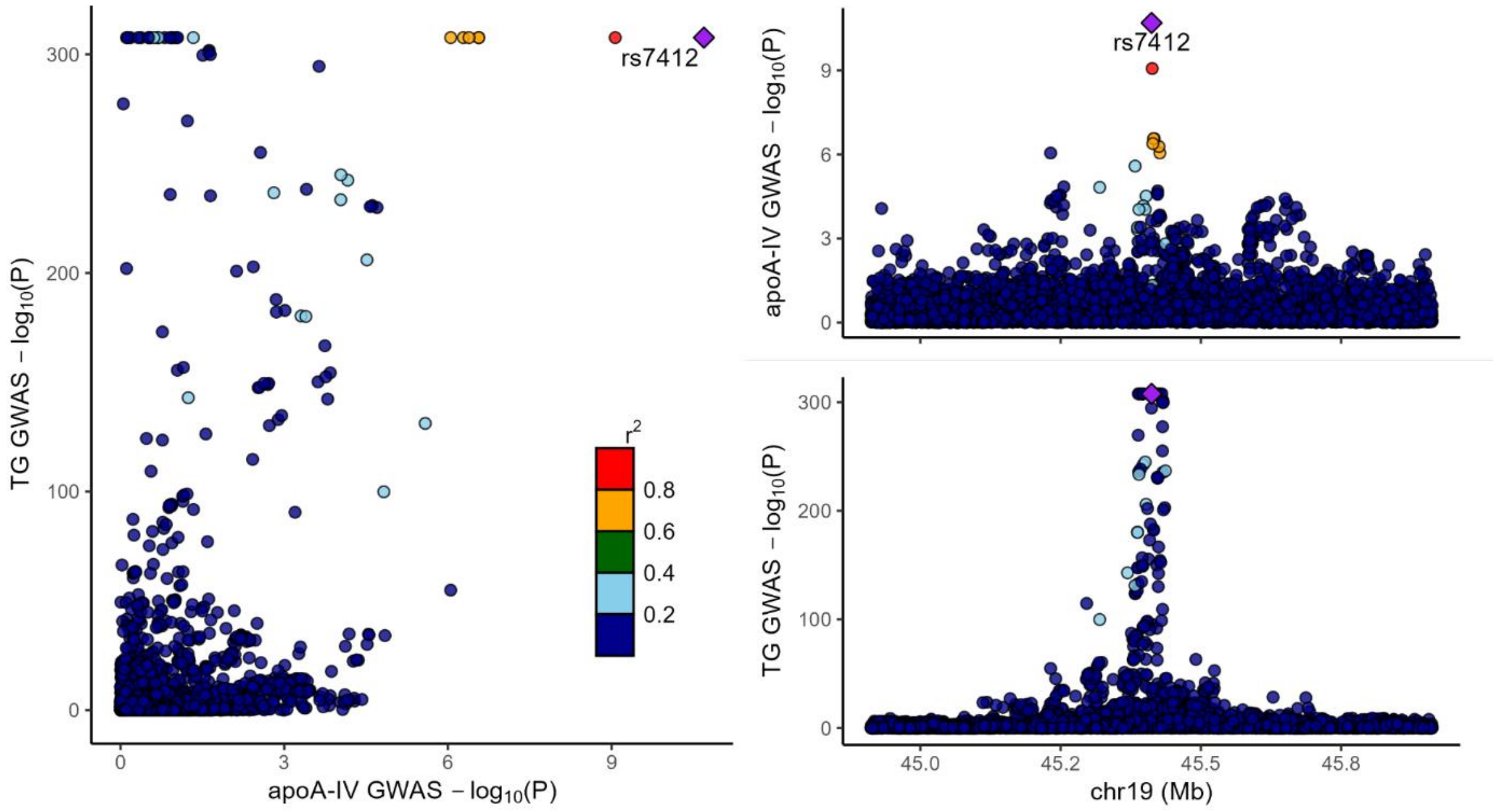

B) GWAS on apoA-IV vs. HDL-C in the *APOA4* region

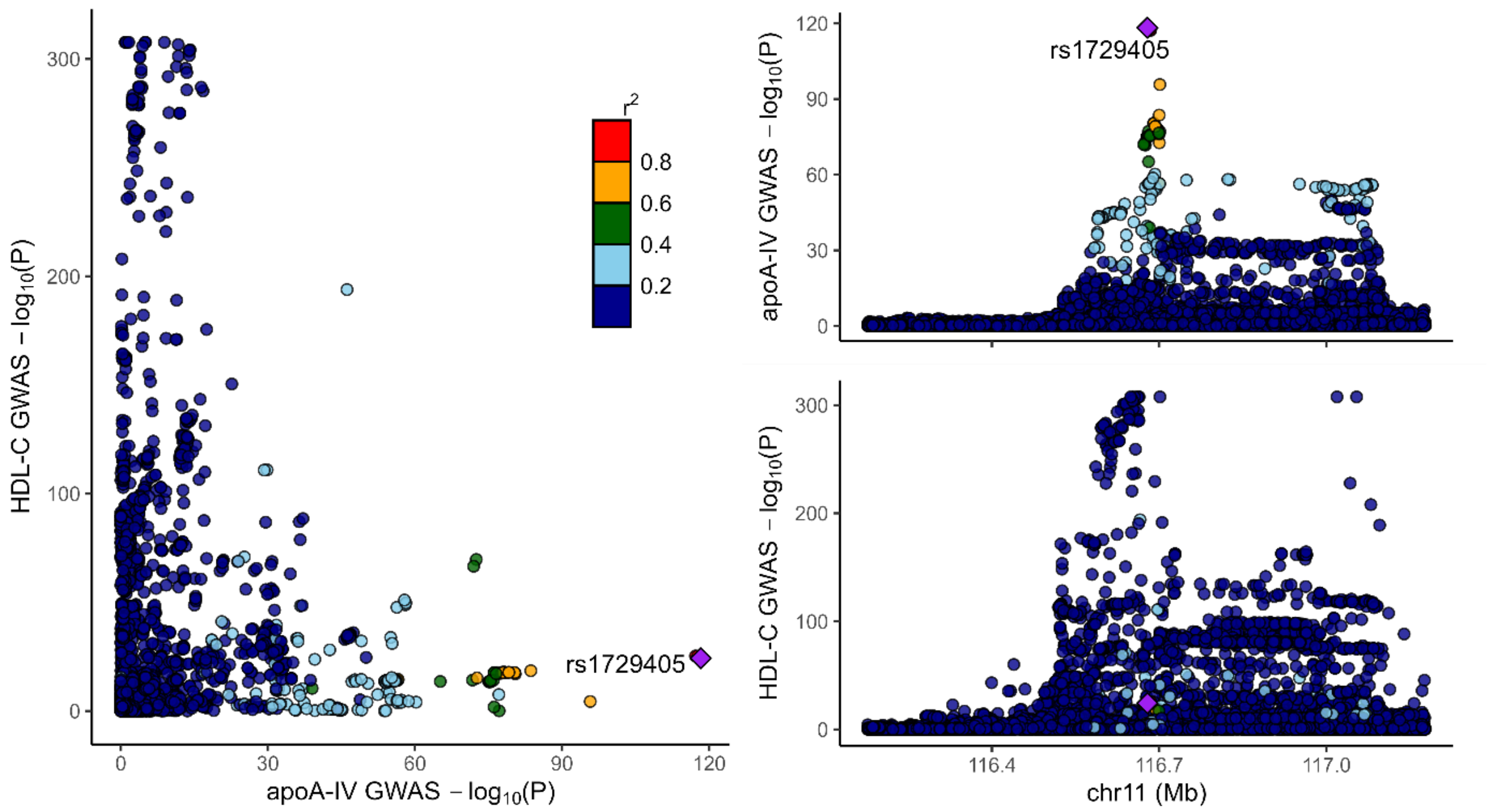

Fig S9: Representative colocalization plots illustrating potential pleiotropy between loci identified in the apoA-IV GWAS and GWAS results from other traits. A) Colocalization at the *APOE* locus between the apoA-IV GWAS (lead SNP: rs7412) and a triglyceride GWAS, showing a shared signal consistent with clear pleiotropy. B) Colocalization at the *APOA4* locus between the apoA-IV GWAS (lead SNP: rs1729405) and an HDL-C GWAS, showing no evidence for main signal colocalization.
